# Predicting Perturbed Human Arm Movements in a Neuro-Musculoskeletal Model to Investigate the Muscular Force Response

**DOI:** 10.3389/fbioe.2020.00308

**Published:** 2020-04-21

**Authors:** Katrin Stollenmaier, Winfried Ilg, Daniel F. B. Haeufle

**Affiliations:** Department of Cognitive Neurology, Hertie Institute for Clinical Brain Research and Werner Reichardt Centre for Integrative Neuroscience, University of Tübingen, Tübingen, Germany

**Keywords:** musculo-skeletal model, motor control, mechanical perturbations, computational model, stretch reflex, internal forces

## Abstract

Human movement is generated by a dynamic interplay between the nervous system, the biomechanical structures, and the environment. To investigate this interaction, we propose a neuro-musculoskeletal model of human goal-directed arm movements. Using this model, we simulated static perturbations of the inertia and damping properties of the arm, as well as dynamic torque perturbations for one-degree-of freedom movements around the elbow joint. The controller consists of a feed-forward motor command and feedback based on muscle fiber length and contraction velocity representing short-latency (25 ms) or long-latency (50 ms) stretch reflexes as the first neuronal responses elicited by an external perturbation. To determine the open-loop control signal, we parameterized the control signal resulting in a piecewise constant stimulation over time for each muscle. Interestingly, such an intermittent open-loop signal results in a smooth movement that is close to experimental observations. So, our model can generate the unperturbed point-to-point movement solely by the feed-forward command. The feedback only contributed to the stimulation in perturbed movements. We found that the relative contribution of this feedback is small compared to the feed-forward control and that the characteristics of the musculoskeletal system create an immediate and beneficial reaction to the investigated perturbations. The novelty of these findings is (1) the reproduction of static as well as dynamic perturbation experiments in one neuro-musculoskeletal model with only one set of basic parameters. This allows to investigate the model's neuro-muscular response to the perturbations that—at least to some degree—represent stereotypical interactions with the environment; (2) the demonstration that in feed-forward driven movements the muscle characteristics generate a mechanical response with zero-time delay which helps to compensate for the perturbations; (3) that this model provides enough biomechanical detail to allow for the prediction of internal forces, including joint loads and muscle-bone contact forces which are relevant in ergonomics and for the development of assistive devices but cannot be observed in experiments.

## 1. Introduction

Humans generate goal-directed movement by an interplay between the nervous system, the biomechanical structures, and the environment, where high-level motor control is fine-tuned to the dynamics of the low-level muscular system and exploits its characteristics (Scott, 2004). Understanding and predicting this dynamic interplay by means of a computational model is relevant for two reasons: firstly, it allows gaining insight into the hierarchical structure of motor control and the sensorimotor integration of muscle-tendon dynamics and reflexes to control (Berniker et al., 2009; Campos and Calado, 2009; Latash, 2010; Kistemaker et al., 2013). Secondly, it provides the opportunity to study internal forces in the musculoskeletal system which are relevant in ergonomics and for the development of assistive devices and otherwise experimentally not accessible (Holzbaur et al., 2005; Pennestrì et al., 2007).

To this end, we here propose a model of human goal-directed arm movements which fulfills the following criteria: (a) it represents the biomechanical structures to a level of detail which allows the prediction of internal joint loads and muscle-bone contact forces; (b) it considers muscle-tendon based short- or long-latency reflexes as the first neuronal responses elicited by an external perturbation; (c) it reproduces experimentally observed responses to static as well as dynamic external perturbation forces which allow to investigate the model's neuro-muscular response and—at least to some degree—represent stereotypical interactions with the environment.

Individually, these criteria have been fulfilled in models before. For criterion (a), models typically consider muscle fiber characteristics (Hill-type muscle models, e.g., Millard et al., 2013; Haeufle et al., 2014b; Siebert and Rode, 2014), tendon non-linear elasticity, neuro-muscular activation dynamics (e.g., Hatze, 1977; Rockenfeller et al., 2015), antagonistic setup (e.g., Schmitt et al., 2019), and anatomical muscle routing (e.g., Holzbaur et al., 2005; Hammer et al., 2019). Such models are used for ergonomics or for the development of assistive devices, but, to our knowledge, do not fulfill at least one of the other two criteria (Holzbaur et al., 2005; Chadwick et al., 2009; Loeb, 2012; Glenday et al., 2019).

Musculoskeletal models which fulfill criterion (b) have also been developed (e.g., Gribble and Ostry, 2000; Kistemaker et al., 2006; Lan and Zhu, 2007; Bayer et al., 2017, review: Todorov, 2004). Two studies further employed perturbations to demonstrate the benefit of combining muscle spindle and Golgi tendon organ signals (Kistemaker et al., 2013) and the role of muscular characteristics in stabilization against different perturbations (Pinter et al., 2012). However, none of these models fulfills criterion (a) as they do not account for anatomical muscle routing. Furthermore, although the latter two studies investigate the reaction to perturbations, they do not fulfill criterion (c): they employ the perturbations to investigate their research questions, but they do not compare their perturbation response to experimental data (Pinter et al., 2012; Kistemaker et al., 2013).

Finally, many models successfully reproduce data from perturbation experiments [criterion (c), reviews see Wolpert and Ghahramani, 2000; Campos and Calado, 2009]. Examples are the predicted response to static perturbations mimicking changes in inertia or damping (Bhanpuri et al., 2014), or to dynamic torque perturbations (Kalveram et al., 2005). Both models incorporate feedback but have no representation of the muscles. Furthermore, they consider feedback on the joint level and not on the muscular level required to investigate sensorimotor integration. In addition to that, none of these models represent the muscular characteristics to fulfill criterion (a).

The purpose of this study was to develop a neuro-musculoskeletal model that fulfills all three criteria. The approach results in a neuro-musculoskeletal model that shows valid responses to both static and dynamic perturbations as reported in the literature (Kalveram and Seyfarth, 2009; Bhanpuri et al., 2014). These responses match those of previous motor control models but allow a novel interpretation of the relative contribution of feedback and biomechanical characteristics as well as the calculation of internal forces. This contribution is a step in the attempt to foster the dual use of musculoskeletal models as tools to study motor control models and as tools for the development of a virtual design and testbed for ergonomics or assistive devices.

## 2. Methods

**Figure 1:**
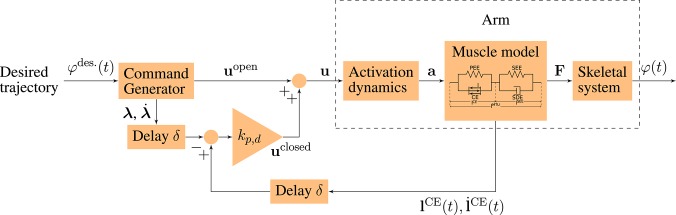
Schematic diagram of the neuro-musculoskeletal model. The desired trajectory φ^des.^(*t*) is a minimum jerk trajectory between a desired starting and an ending point. The command generator maps this trajectory to an open-loop motor command **u**^open^ and to desired muscle fiber lengths and contraction velocities (**λ**, $\overset{∙}{\lambda }$) that correspond to the desired trajectory. The total motor command **u** is fed into the model of the activation dynamics of muscles which relates the neuronal stimulation **u** to muscular activity **a** that drives the muscle model. The muscles produce forces **F** that act on the skeletal system resulting in a simulated movement φ(*t*) of the arm. In the time-delayed feedback loop, the sensory system which represents a simplified version of the muscle spindles measures the current lengths and contraction velocities of the muscle fibers (${\text{l}}^{\text{CE}}\left(t\right),{\overset{∙}{\text{l}}}^{\text{CE}}\left(t\right)$). They are compared to the desired values (**λ**, $\overset{∙}{\lambda }$) and the resulting feedback error is multiplied by the feedback gains *k*_*p*_ and *k*_*d*_ (see Equation 4).

In order to simulate goal-directed arm movements, we combine a musculoskeletal model of the arm including two degrees of freedom and six muscles (based on Kistemaker et al., 2007; Suissa, 2017; Driess et al., 2018) with a neuronal control model (based on the concept of Bhanpuri et al., 2014). Both parts are described in more detail in the following. The structure of the neuro-musculoskeletal model is illustrated in Figure 1.

**Figure 2:**
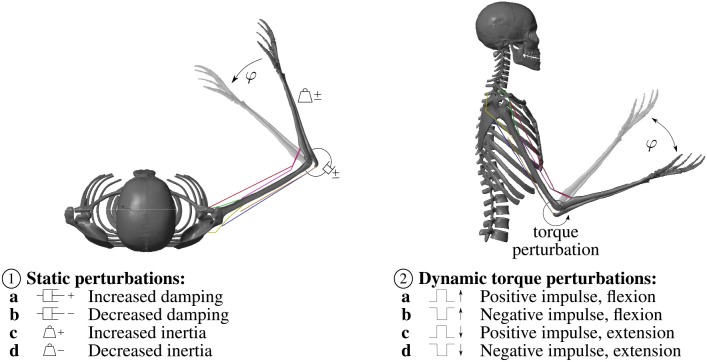
Overview over the applied static and dynamic perturbations. ① The static perturbations of the inertia and viscosity properties of the arm during a flexion movement in the horizontal plane (without gravity) are: **a** Increased damping (+0.30Nms rad^−1^) **b** Decreased damping (−0.31N m s rad^−1^) **c** Increased inertia (+0.039kg ms^2^) **d** Decreased inertia (−0.032kg ms^2^), in accordance with Bhanpuri et al. (2014). ② During the dynamic torque perturbations a constant torque that mimics gravity (−1.5Nm) is applied. Hence, we visualized this movement as a movement in the vertical plane. The perturbation is a temporal torque impulse in or against the direction of movement: **a** Positive torque impulse (+5Nm) during a flexion movement **b** Negative torque impulse (−5Nm) during a flexion movement **c** Positive torque impulse (+5Nm) during an extension movement **d** Negative torque impulse (-5Nm) during an extension movement, in accordance with Kalveram et al. (2005).

To investigate the model's interaction with the environment and compare it to experimental results, static perturbations of the inertia and viscosity properties of the arm (as reported in Bhanpuri et al., 2014) as well as dynamic torque perturbations (as reported in Kalveram et al., 2005) are applied. An overview over the applied perturbations is given in Figure 2.

### 2.1. Musculoskeletal Model of the Arm

The musculoskeletal model *Arm26* (2 degrees of freedom, six muscles, see Bayer et al., 2017; Driess et al., 2018) of the human arm is described in detail in the Supplementary Material. The arm model consists of two rigid bodies (lower and upper arm) that are connected via two one-degree-of-freedom revolute joints that represent the shoulder (glenohumeral) and elbow joint (see Figure 1, within dashed box for schematics and Figure 2 for a visualization). Active forces are generated by six muscle-tendon units (MTUs, see Figure 2), four monoarticular (shoulder anteversion, shoulder retroversion, elbow flexor, elbow extensor) and two biarticular muscles (biarticular flexor, biarticular extensor). The muscles are stimulated by a neuronal control stimulation signal **u**. The model of the activation dynamics predicts the activity **a** of the muscle depending on the current muscle stimulation, considering the fiber length dependency (Hatze, 1977) (see Figure 1). Depending on the muscular activity, the force of each MTU is modeled using a Hill-type model accounting for force-length-velocity characteristics, tendon and parallel tissue elasticity, and damping in the tendon (Haeufle et al., 2014b). Muscle path geometry, i.e., origin, insertion and path deflection, is implemented to match experimental lever arm data. For the joint angle-dependent deflection geometry, we used the via-ellipse approach confining the path of the muscle to geometric ellipses attached to the rigid bones (Hammer et al., 2019). This algorithm allows to calculate muscle-bone contact forces and applies forces to the bones such that internal joint loadings can be predicted.

The parameters used in the models are not subject-specific but represent a generic man and are collected from different sources (among others: van Soest et al., 1993; Kistemaker et al., 2006; Mörl et al., 2012; Bhanpuri et al., 2014) that are listed in detail in the Supplementary Material. Due to the muscle-tendon model in combination with anatomical muscle routing, our model provides the necessary level of biomechanical detail to determine internal muscular and joint loads as well as muscle-bone contact forces. Hence, criterion (a) that we established in the introduction is fulfilled.

The experimental perturbations that we reproduce in this simulation study were confined to the elbow joint. Thus, we here fix the shoulder joint to 30° such that only one-degree-of-freedom movements are possible. Hence, the monoarticular shoulder muscles have no effect on the movement and are excluded from our investigations. To make the results comparable to experiments, the inertia properties of the forearm were changed according to an arm that is attached to an exoskeleton robot that was used by Bhanpuri et al. (2014).

### 2.2. Control Model

The neuronal control model is illustrated in Figure 1. It is based on the control model that was proposed by Bhanpuri et al. (2014) to reproduce static perturbations in a torque-driven model of the arm. The input to the controller is a desired trajectory φ^des.^(*t*) that is considered to be a result of the movement planning. The controller consists of an open-loop command **u**^open^ and a closed-loop signal **u**^closed^ that incorporates proprioceptive feedback. The total stimulation *u*_*i*_ is the sum of those components and represents α-motor neuron activity. For each muscle *i*, it is calculated as

(1) $$\begin{array}{l} u_{i}\left(t\right):={\left\{u_{i}^{\text{open}}\left(t\right)+u_{i}^{\text{closed}}\left(t\right)\right\}}_{0}^{1}, \end{array}$$

where the operation ${\left\{x\right\}}_{0}^{1}$ sets values *x* < 0 to 0 and *x* > 1 to 1.

The total motor command ${\left\{u_{i}\left(t\right)\right\}}_{i=1}^{6}$ is fed into the musculoskeletal model resulting in a simulated movement φ(*t*) of the arm. This control approach can be classified as a modified hybrid equilibrium point (EP) controller where the open-loop signal is intermittent while the feedback signal is continuous (see Kistemaker et al., 2006).

#### 2.2.1. Movement Planning

We assume that a higher-level structure conducts planning of the movement and provides a desired kinematic movement trajectory φ^des.^(*t*) as an input to the lower-level control structures that are modeled here. Therefore, the input to our controller is the desired trajectory which we determined by generating a minimum-jerk trajectory between desired starting and ending angles. To this end, a fifth-order polynomial approach for the desired angle trajectory φ^des.^(*t*) is chosen in accordance with Flash and Hogan (1985) who have shown that their mathematical model shows the typical bell-shaped velocity profile and predicts experimental observations of voluntary unconstrained point-to-point movements in a horizontal plane.

#### 2.2.2. Open-Loop Control Generates Reference Trajectory

The command generator maps the desired trajectory φ^des.^(*t*) to an open-loop motor command **u**^open^, and to desired contractile element lengths and velocities (**λ**, $\overset{∙}{\lambda }$) that correspond to the desired trajectory. Using a musculoskeletal model, the generation of these motor commands is non-trivial since the system is redundant (degree of freedom problem, see Bernstein, 1967; Shadmehr, 1991) and non-linear. In addition to that, the fact that the activation dynamics and the muscle model are described by first-order differential equations including time delays and the resulting time-dependency prohibits the straight-forward calculation of the inverse problem.

**Figure 3:**
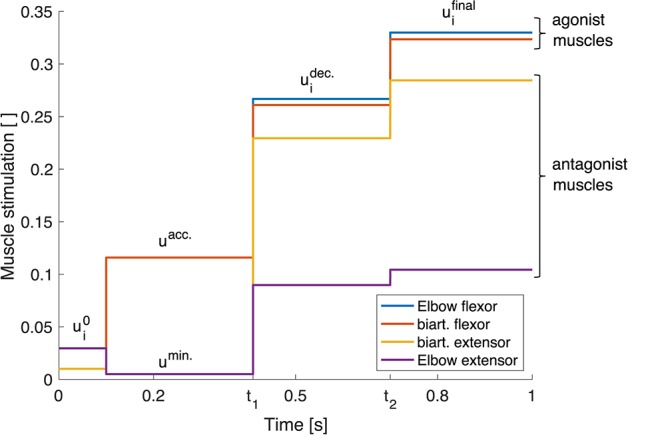
Triphasic stimulation pattern for a flexion movement. Starting from the initial position at *t* = 0.1 *s*, during the acceleration phase, mainly the agonist muscles are active. In the second phase between *t* = *t*_1_ and *t* = *t*_2_, both muscle groups are active, braking the movement. In the last phase for *t* > *t*_2_, again both muscle groups are active in order to reach the final position and hold it with a desired level of co-contraction.

To simplify this process, instead of deriving a continuous set of stimulations over time, we introduce a triphasic stimulation pattern with a limited number of parameters (see Equation 2, illustrated in Figure 3). It is inspired by the three phases that have been observed in muscle surface electromyogram (EMG) patterns during fast point-to-point movements (see e.g., Wachholder and Altenburger, 1926; Wierzbicka et al., 1986; Kistemaker et al., 2006): an acceleration phase where mostly the agonist muscles are active which is followed by a braking phase and a final phase which keeps the arm in the desired final position. Hence, the muscles are divided into two groups: the agonists and the antagonists for a movement. We define the muscle stimulations over time for those muscle groups as

(2) $$\begin{array}{l} u_{i}^{\text{open}}\left(t\right):=\{\begin{array}{cc} u_{i}^{0} & \text{for }t<\text{0.1}\;\text{s}\\ u_{i}^{\text{acc.}}=\{\begin{array}{cc} u^{\text{acc.}} & \text{for agonist muscles}\\ u^{\text{min.}} & \text{for antagonist muscles} \end{array} & \text{for }\text{0.1}\;\text{s}\leq t<t_{1}\\ u_{i}^{\text{dec.}} & \text{for }t_{1}\leq t<t_{2}\\ u_{i}^{\text{final}} & \text{for }t_{2}\leq t. \end{array} \end{array}$$

Following this approach, the control parameters that are required to follow the desired trajectory need to be determined.

The initial and the final position are determined to be stable equilibrium positions, i.e.,

(3) $$\begin{array}{l} \overset{∙}{\varphi }=0\;\text{and }\ddot{\varphi }=0, \end{array}$$

which leads to the condition that the net joint moment vanishes in these positions. This allows for the determination of the necessary muscle stimulations $u_{i}^{0}$ and $u_{i}^{\text{final}}$ to hold the initial and the final position by minimizing $\overset{4}{\underset{i=1}{\sum }}\left(u_{i}-u^{\text{des.}}\right)$ subject to the constraint that the sum of all torques acting on a joint is zero, i.e., the system is in a stable equilibrium position. Herein, the desired level of stimulation *u*^des.^ allows influencing the level of co-contraction. The condition that the system is supposed to be in equilibrium at *t* = 0 defines the initial conditions. The final phase starts at *t*_2_ = 0.7 s which is approximately the time when the final position is reached.

The dynamic movement between those equilibrium positions (0.1 s < *t* < *t*_2_) is parametrized such that it is close to the desired trajectory φ^des.^(*t*):

In the acceleration phase, the muscle stimulation *u*^acc.^ and the switching time *t*_1_ are optimized using a Bayesian optimization approach (see e.g., Brochu et al., 2010) where the squared point-wise difference between the current trajectory and the desired trajectory is minimized. The minimal level of stimulation *u*^min.^ is set to a fixed value (0.005) in order to reduce the search space for possible stimulations.

The muscle stimulation pattern $u_{i}^{\text{dec.}}$ in the braking phase is determined analogously to the stimulations $u_{i}^{\text{final}}$ but with a lower level of co-contraction to reach the final position following the desired pathway.

In the following, these optimized muscle stimulation patterns are used as open-loop signals $u_{i}^{\text{open}}\left(t\right)$. If no external perturbation occurs, this stimulation pattern generates a trajectory that is close to the desired minimum jerk trajectory φ^des.^(*t*). This trajectory will be used as reference hereafter.

#### 2.2.3. Closed-Loop Response to Perturbations

If a perturbation occurs, the movement trajectory changes. As a consequence, the actual fiber lengths and contraction velocities differ from the values from the reference trajectory. In this case, the feedback loop modifies the control signal (see Equation 1). This proprioceptive feedback is incorporated in the closed-loop signal $u_{i}^{\text{closed}}\left(t\right)$ by comparing the actual lengths and contraction velocities (${\text{l}}^{\text{CE}}\left(t\right),{\overset{∙}{\text{l}}}^{\text{CE}}\left(t\right)$) of the muscle fibers (contractile elements, CEs) of the muscles to desired values (**λ**(*t*), $\overset{∙}{\lambda }\left(t\right)$). The desired CE lengths and velocities (**λ**, $\overset{∙}{\lambda }$) are set to the values (${\text{l}}^{\text{CE}}\left(t\right),{\overset{∙}{\text{l}}}^{\text{CE}}\left(t\right)$) recorded during an unperturbed movement. So, as long as there is no external perturbation, the feedback error is zero and hence the closed-loop signal vanishes.

Since the information about the current state of the muscle only becomes available with a neuronal delay, a time lag δ is introduced. To investigate different hierarchy levels of feedback mechanisms, we tested both, a short-latency and a long-latency stretch reflex. For the short-latency response, the time delay is set to 25 ms in accordance with similar arm models (Gribble et al., 1998; Kistemaker et al., 2006; Bayer et al., 2017) which is in a physiologically plausible range [R1 response (Pruszynski et al., 2011; Kurtzer et al., 2014; Scott, 2016; Weiler et al., 2016)]. This short-latency feedback represents a simplified model of the spinal, mono-synaptic muscle spindle reflex (Pruszynski and Scott, 2012; Weiler et al., 2019), assuming that the muscle spindles provide accurate time-delayed information about the muscle fiber lengths and contraction velocities (Kistemaker et al., 2006). Since experimental findings indicate that the long-latency stretch reflex plays an important role in the reaction to mechanical perturbations in goal-directed reaching movements (e.g., Kurtzer et al., 2014; Weiler et al., 2016), we also implemented a long-latency feedback loop by setting the time delay to 50 ms [R2 response (Pruszynski et al., 2011; Scott, 2016)]. Since both, short- and long-latency feedback are implemented with the same mathematical model (see below) and lead to similar results, we focus in the following on the long-latency response, while the short-latency responses to the perturbations can be found in the Supplementary Material. By considering these muscle-tendon based reflexes, our model fulfills criterion (b) that we suggested in the introduction.

The closed-loop signal $u_{i}^{\text{closed}}\left(t\right)$ for each muscle *i* is calculated as

(4) $$\begin{array}{lll} u_{i}^{\text{closed}}\left(t\right) & := & \frac{k_{p}}{l^{\text{CE},\text{opt}}}\left(l_{i}^{\text{CE}}\left(t-\delta \right)-\lambda_{i}\left(t-\delta \right)\right)\\ \;+ & \frac{k_{d}}{l^{\text{CE},\text{opt}}}\left({\overset{∙}{l}}_{i}^{\text{CE}}\left(t-\delta \right)-{\overset{∙}{\lambda }}_{i}\left(t-\delta \right)\right), \end{array}$$

where *k*_*p*_ and *k*_*d*_ are the feedback gains and *l*^CE,opt^ stands for the optimal length of the contractile element. The feedback gains *k*_*p*_ and *k*_*d*_ as well as the desired level of co-contraction in the braking phase *u*^des.,dec.^ play an important role in the way how the system reacts to perturbations. Therefore, they are optimized in order to reproduce the answer to all four static perturbations seen in experiments.

**Figure 4:**
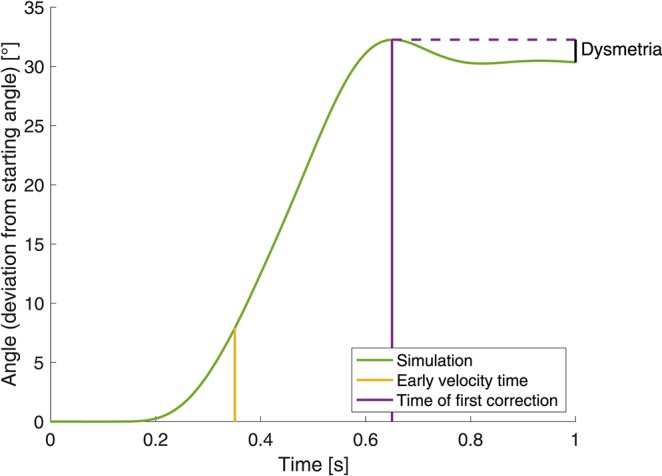
Illustration of the determination of early velocity and dysmetria.

In the objective function for this optimization, we incorporated the quantities early velocity and dysmetria (as used by Bhanpuri et al., 2014, illustrated in Figure 4) that we also use as evaluation criteria for the static perturbations below. *Early velocity* is defined as the joint angle velocity 155 ms after the first time the velocity exceeds 10 °/s. *Dysmetria* is defined as the difference between the final position (at t=1 s) and the position at the time of first correction. Herein, the time of first correction is the time when the absolute value of the angular velocity is smaller than 2 °/s or the absolute value of the angular acceleration falls below 2 °/s^2^. The objective function is minimized using the pattern search algorithm in Matlab® and is defined as

(5) $$\begin{array}{l} \sum_{\begin{array}{c} \text{static}\\ \text{perturbation types} \end{array}}[{\left(\frac{\text{early velocity difference simulation - mean early velocity difference experiment}}{\text{maximal standard deviation early velocity difference experiment}}\right)}^{2}\\ \;+{\left(\frac{\text{dysmetria difference simulation - mean dysmetria difference experiment}}{\text{maximal standard deviation dysmetria difference experiment}}\right)}^{2}]\\ =\sum_{\begin{array}{c} \text{static}\\ \text{perturbation types} \end{array}}\left[{\left(\frac{\Delta v_{0}^{\text{sim}}-\mu (\Delta v_{0}^{\text{exp}})}{\max\sigma (\Delta v_{0}^{\text{exp}})}\right)}^{2}+{\left(\frac{\Delta d^{\text{sim}}-\mu (\Delta d^{\text{exp}})}{\max\sigma (\Delta d^{\text{exp}})}\right)}^{2}\right], \end{array}$$

with $v_{0}^{\text{sim}}$: early velocity, *d*: dysmetria, μ: mean, σ: standard deviation, Δ: difference that is calculated as the early velocity/dysmetria of the perturbed movement minus the early velocity/dysmetria of the reference movement.

The whole set of resulting control parameters can be found in Table 2 in the Appendix. To quantify the influence of these control parameters on the resulting movements, we performed a sensitivity analysis (see Appendix).

### 2.3. Simulation Experiments

To test whether this model also fulfills criterion (c) from the introduction, we simulated its response to static and dynamic perturbations.

#### 2.3.1. Static Perturbation of Inertia and Viscosity

Bhanpuri et al. (2014) performed experiments where healthy subjects carried out goal-directed single-joint arm movements while the arm was attached to an exoskeleton robot. Each subject performed two blocks with 40 trials each of which 36 trials were null trials (without perturbation). In the perturbation trials, the robot exerted a force to mimic changes in the dynamic properties of the arm, in particular inertia and viscosity. The movements were performed in a horizontal plane (Figure 2 ①).

In our computer simulation, we adapted the moment of inertia of the modeled forearm to account for the influence of the robot arm to be able to compare our simulation results to their experiments. In accordance with Bhanpuri et al. (2014), the static perturbations were an increase in moment of inertia (+0.039kgms^2^), a decrease in inertia (−0.032kgms^2^), an increase in damping (+0.30Nms rad^−1^) or a decrease in damping (−0.31Nms rad^−1^) (Figure 2 ①).

**Evaluation criterion**: In order to compare the simulation results to the experimental data, we introduced an evaluation criterion as used by Bhanpuri et al. (2014). They investigate the relation between early velocity and dysmetria, as defined above in section 2.2.3 and illustrated in Figure 4.

#### 2.3.2. Dynamic Torque Perturbation

In analogy with the experiments described in Kalveram et al. (2005) and Kalveram and Seyfarth (2009), a dynamic torque perturbation was applied to the simulated pointing movement (Figure 2 ②). A constant torque that mimics gravity (−1.5 N m) is applied. The perturbation is an additional temporal torque change in or against the direction of movement (±5 N m). The perturbation starts after 25% of the movement (corresponds to 7.5° of 30° in total) and lasts 37.5 ms. Hence, relative to the total movement, we apply the same perturbation as Kalveram et al. (2005). The starting and final position and all other biomechanical and control parameters are identical to the static perturbation simulations ①.

**Evaluation criterion**: For the dynamic torque perturbation, we chose the quotient of the angular velocity at the elbow joint at the beginning and at the end the perturbation as an evaluation criterion:

(6) $$\begin{array}{l} \text{velocity quotient}:=\frac{\text{angular velocity at the beginning of the perturbation}}{\text{angular velocity at}\Delta t\text{ after the beginning of the perturbation}}. \end{array}$$

Setting Δ*t* to the duration of the perturbation (37.5 ms), the velocity quotient relates the angular velocity at the beginning of the perturbation to the one at the end of the perturbation. This allows investigating the muscle-dominated response to the perturbations. In addition to that, we also evaluate the velocity quotient of the angular velocity at Δ*t* = 100 ms after the beginning of the perturbation, which quantifies also the first neuronal response.

#### 2.3.3. Implementation

The arm model and the optimization and analysis scripts are implemented using Matlab®/Simulink® version 2018a with the Simscape Multibody™environment. For all simulations, the variable-step Matlab ODE solver *ode15s* with relative solver tolerance 1 × 10^−5^ has been used. The absolute tolerance and the minimum/maximum/initial step size are set to be determined automatically.

For comparison, the experimental results were digitized from Kalveram and Seyfarth (2009) and Bhanpuri et al. (2014). For a smooth appearance and for the calculation of the angular velocity, we fitted a smoothing spline to the digitized discrete data (using the curve fitting toolbox in Matlab®).

### 2.4. Open-Loop and Torque-Driven Model as Comparison

To investigate the influence of the implemented feedback mechanism, we applied the same perturbations to an open-loop controlled version of our model, i.e., without the implemented feedback loop (*k*_*p*_ = 0 and *k*_*d*_ = 0).

In addition to that, we implemented an idealized torque-driven model to compare the reaction to external forces to those of the musculoskeletal model. This comparison allows investigating the contribution of the visco-elastic reaction forces which are generated by the muscle-tendon contraction dynamics (*preflex* forces). The torque-driven model uses the same mechanical parameters (segment lengths, masses, inertia) as the musculoskeletal model. To determine the torque that is necessary to reproduce the musculoskeletal model's movement, we recorded the net joint torque that is applied by the muscles during both the unperturbed movement. In accordance with the model of Bhanpuri et al. (2014), the feedback is based on the joint positions with a delay of 100 ms representing a long-latency reflex.

## 3. Results

We here show the results for the long-latency feedback loop (50 ms delay). The short-latency responses (25 ms delay) to the perturbations is quite similar and can be found in the Supplementary Material.

### 3.1. Intermittent Open-Loop Signals Reproduce Unperturbed Movement

**Figure 5:**
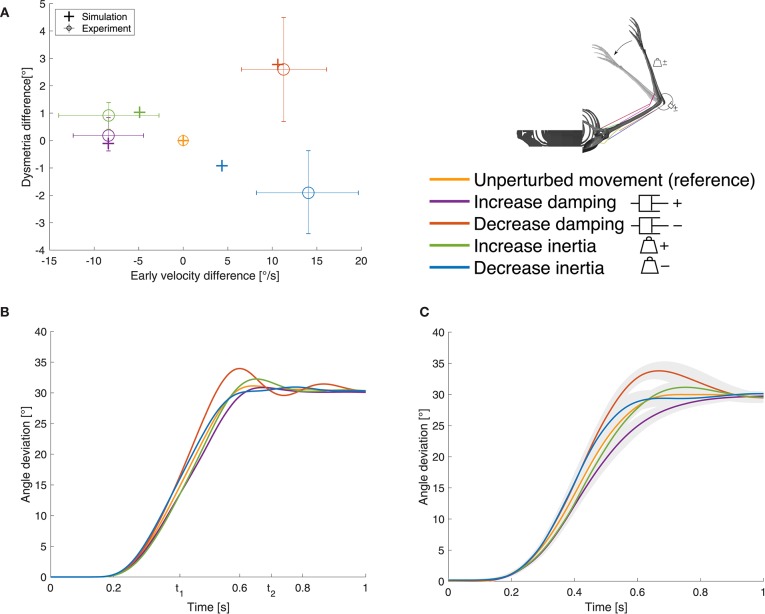
Results for case ①. **(A)** Evaluation criterion for the static perturbations: early velocity difference in relation to the dysmetria difference (both calculated as the early velocity/dysmetria of the perturbed movement minus the early velocity/dysmetria of the reference movement) shown for both, simulation and experiment. The experimental results are digitized from Bhanpuri et al. (2014), the control group averages (*n* = 11) are shown and the error bars indicate standard deviation. **(B)** Our simulation results and **(C)** experimental results digitized from Bhanpuri et al. (2014) for one typical control subject in null condition (reference) and with perturbations (shaded areas indicate standard deviation).

The simulation of the unperturbed movement is in good agreement with the desired minimum jerk trajectory and with the experimental data (see Figures 5, **7**, orange curves). As mentioned above, without perturbations the feedback signal vanishes. So, the movement is solely controlled by the open-loop command which is a piecewise constant function over time. This unperturbed movement is the reference for the perturbed cases.

### 3.2. Static Perturbation of Inertia and Viscosity

In presence of the static perturbations, the simulation and experimental results show the same qualitative behavior in the relation between early velocity difference and dysmetria difference (Figure 5A). An increase in inertia leads to a lower early velocity which results in higher dysmetria. A decrease in inertia causes an increase in early velocity which leads to lower dysmetria. For the damping perturbations, it is the other way round. The comparison of the movement trajectory in the simulation (Figure 5B) and the experiments of Bhanpuri et al. (2014) (Figure 5C) shows a qualitatively and quantitatively similar behavior at the beginning of the movement. Toward the end of the movement, the subject in the experiment tends to take longer to reach the final position, especially for the damping perturbations. Note that we only compared our results with experimental trajectories of one typical control subject and with early velocity/dysmetria difference of a small control group, respectively, while we used generic, not subject-specific parameters for the mechanical description of the limb.

**Figure 6:**
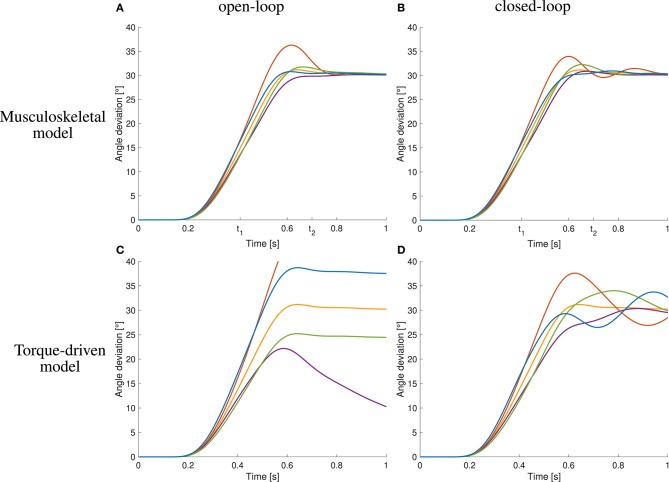
Comparison to open-loop and torque-driven model for case ①. **(A)** Resulting trajectories when controlling the musculoskeletal model open-loop, **(B)** trajectories when controlling the musculoskeletal model closed-loop, **(C)** trajectories when controlling a purely torque-driven model open-loop, and **(D)** trajectories when controlling a purely torque-driven model closed-loop.

The open-loop controlled system shows a similar response to the static perturbations as the closed-loop version and also as the subjects in the experiment (Figure 6A). However, in three of four cases, the closed-loop controller leads to better results than the open-loop approach and also the sum of all cases is smaller (Figure 6A vs. Figure 6B and Table 1). The only case that does not profit from the feedback and leads to similar results is the decreasing of inertia.

**Table 1:** Quantification of the difference between simulation and experiment for case ① by evaluating the cost function (Equation 5) that was used in the optimization of the closed-loop control parameters and splitting it into the contributions of the different perturbations.

	**Closed-loop**	**Open-loop**
Increased damping	0.19	4.43
Decreased damping	0.03	1.77
Increased inertia	0.43	0.69
Decreased inertia	3.19	2.87
Sum of all cases	3.84	9.76

*For the single cases, a value smaller than one means that the result lies within the experimental standard deviation area (taking the maximum standard deviation in each direction)*.

The trajectories generated by the torque-driven model do not reach the desired target position without feedback (Figure 6C). With feedback, the trajectories get closer to what has been observed in the experiments, but there are oscillations around the target position (Figure 6D).

An increase in arm inertia causes an overshoot of the movement using the musculoskeletal model with and without feedback while the forward-controlled torque model predicts an undershoot (Figure 6). The former counter-intuitive behavior was also observed in the experiments (Figure 5C).

### 3.3. Dynamic Torque Perturbation

**Figure 7:**
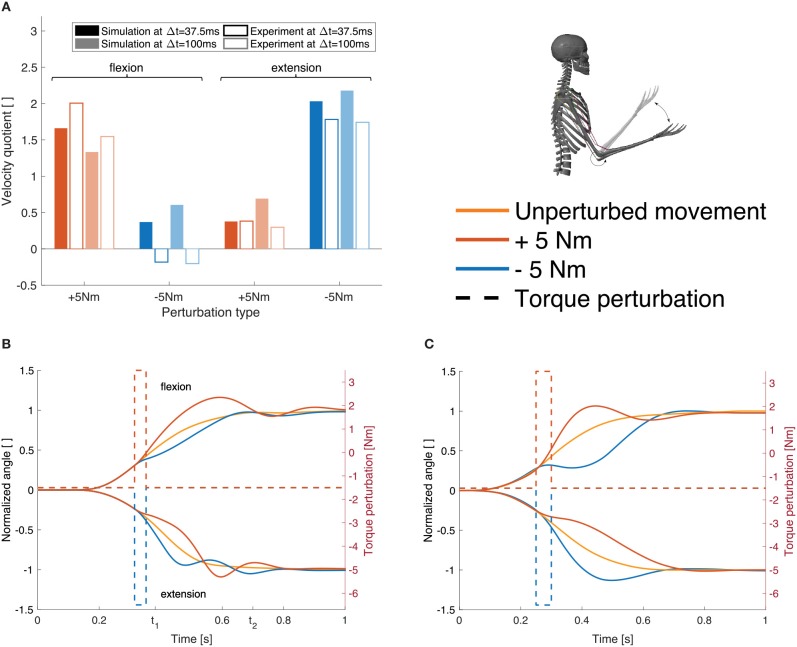
Results for case ②. **(A)** Evaluation criterion for the dynamic perturbations: the quotient of the angular velocity at the beginning of the perturbation and after Δ*t* (37.5 and 100 ms, see Equation 6) shown for both, the simulation results (filled bars) and the experimental results (empty bars) for all four perturbation types (experimental results are digitized from Kalveram and Seyfarth, 2009). **(B)** Joint angle trajectories for the four different perturbation types in our simulation and **(C)** in the experiment (digitized from Kalveram and Seyfarth, 2009). Note that the experimental results show the trajectory for one typical control subject. The upper curves show flexion movements, the lower curves show extension movements. The dashed lines visualize the applied torque perturbations.

The response to the dynamic perturbations in the simulation is qualitatively similar to what has been observed in the experiments (Figures 7B,C). Most relevant here is the reaction directly after the perturbation which reflects in a change in angular velocity. Therefore, we calculated the relation between the angular velocity in the elbow joint at the beginning and the end of the perturbation (Figure 7A, Δ*t*= 37.5 ms). For a perturbation in the direction of the movement, the velocity is approximately doubled while it is halved for perturbations against the direction of movement. The velocity quotient between the velocity in the beginning and the one 100 ms after the beginning of the perturbation (Figure 7A, Δ*t*= 100 ms) deviates more from the experiment than the one after 37.5 ms.

Note that no parameters were tuned to match the perturbed trajectories. For all static and dynamic perturbation types, the same feedback gains, delays and desired levels of co-contraction are used. For case ②, some parameters need to be re-optimized in comparison to ① to compensate for the constant torque that mimics gravity and to allow for an extension movement. The whole set of control parameters can be found in Table 2 in the Appendix.

**Figure 8:**
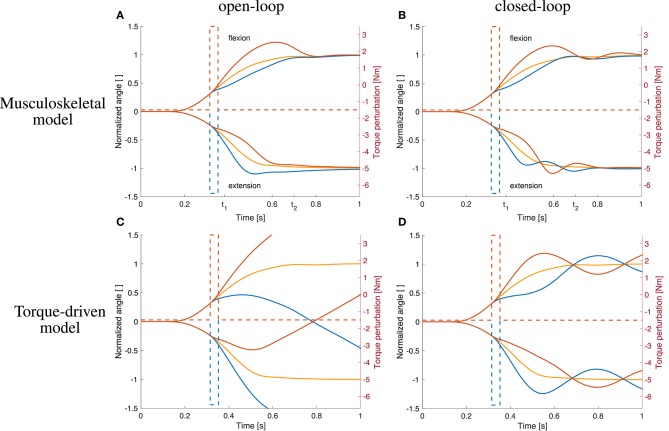
Comparison to open-loop and torque-driven model for case ②. **(A)** Resulting trajectories when controlling the musculoskeletal model open-loop, **(B)** trajectories when controlling the musculoskeletal model closed-loop, **(C)** trajectories when controlling a purely torque-driven model open-loop, and **(D)** trajectories when controlling a purely torque-driven model closed-loop with the same controller as described above.

The open-loop controlled system shows a similar response to the dynamic perturbations as the closed-loop version and also as the subjects in the experiment (Figures 8A,B).

The trajectories generated by the torque-driven model do not reach the desired target position without feedback (Figure 8C). With feedback, the trajectories get closer to what has been observed in the experiments, but there are oscillations around the target position (Figure 8D).

### 3.4. Internal Force Responses

**Figure 9:**
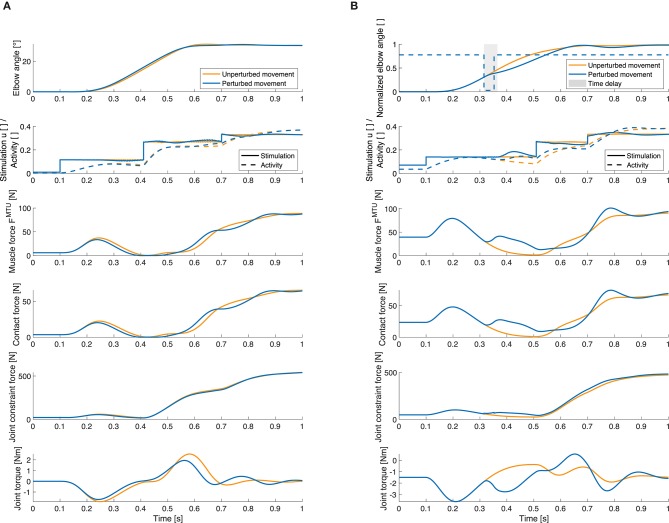
Selection of quantities that can be investigated using our model. Elbow joint angle, muscle stimulation and activity, muscle force, muscle-bone contact force, joint constraint force and active joint torque for the unperturbed trajectory (orange) and for a perturbed movement (blue). These results are exemplary shown for the elbow flexor muscle and **(A)** for an increase in inertia and **(B)** for a flexion movement with a negative torque impulse perturbation. Here, the gray area visualizes the length of the time delay in the controller (50ms), i.e., the time after the perturbation before the feedback mechanism is activated. Note that the total muscle stimulation in the unperturbed case is equal to the open-loop contribution in the perturbed case. For all forces, the resultant force is shown. The contact force is the force at the first deflection ellipse (positions of the ellipses see Supplementary Material). The active joint torque represents the torque acting on the joint that is a consequence of the muscle forces.

Our model approach allows for analyses of internal muscular and joint force responses as well as the proprioceptive feedback signals that cannot be observed in experiments. To show the possibilities this method offers, we evaluated the joint angle, muscle stimulation and resulting activity, internal muscle and joint forces and active joint torque exemplary for one static and one dynamic perturbation case and for one muscle (Figure 9). The changes in the total muscle stimulation are due to the implemented feedback mechanism: For example in Figure 9B, the perturbation acts against the direction of movement, so the muscle stimulation is increased to compensate for it. Also the muscle force is increased as a consequence of the perturbation. In consequence, the contact force and the constraint force in the elbow joint are increased as well.

## 4. Discussion

Our goal was to propose a model of human goal-directed arm movements which fulfills all three criteria that we formulated in the introduction: Our neuro-musculoskeletal model shows valid responses to both static and dynamic perturbations and therefore fulfills criterion (c). This alone is novel, as typically only one category of perturbations is studied and reproduced by previous models. The predicted response to both types of perturbations is an emerging behavior of the sensorimotor integration in the model which was achieved by fulfilling the other two criteria, both specifying the level of detail of the modeling. The high level of biomechanical detail allows predicting muscle-tendon based proprioceptive feedback signals, internal muscle forces, muscle-bone contact forces, and joint loads (Figure 9), all of which require the representation of muscle-tendon complexes and geometrical muscle routing in the model [criterion (a)]. In consequence, kinematic- or torque-based control concepts of human motor control are not applicable, as a control input is required on the muscular level for our model (one for each muscle). The proposed controller is a combination of an open-loop controller and a low-level muscle spindle signal based controller [criterion (b)]. The open-loop controller generates a (close-to) minimum jerk trajectory for the unperturbed movement. Only in the presence of a perturbation, the closed-loop control contributes to the muscle stimulations. Thus, this model allows for gaining insights into the sensorimotor integration in response to external forces.

The experimental data on the applied static (Bhanpuri et al., 2014) and dynamic perturbations (Kalveram and Seyfarth, 2009) that we used to validate our model response has been previously reported in the literature. The static perturbations represent changes in inertia and viscosity continuously affecting the dynamics of the lower arm (Bhanpuri et al., 2014). Such force fields have been a valuable tool to investigate motor control models (e.g., Pinter et al., 2012), and particularly, motor adaptation (e.g., Gribble and Ostry, 2000; Kistemaker et al., 2010, review: Franklin and Wolpert, 2011). Please note that in this contribution we focused on the non-adaptive neuro-muscular response in the sense of a sudden response to an unexpected perturbation in between a large set of null-trials, thus, neglecting motor learning (e.g., Burdet et al., 2006; Yang et al., 2007; Shadmehr et al., 2010). This is also the case for the dynamic perturbations, which represent a sudden and time-limited external torque. These perturbations represent a broad spectrum of systematic perturbations as they may occur in ergonomically relevant scenarios or in the interaction with assistive devices.

Individually, the response to these perturbations have been reproduced by motor control models before [static (Bhanpuri et al., 2014) and dynamic (Kalveram and Seyfarth, 2009)]. Both models reproduced the experimental kinematics by means of a torque in the elbow joint. Both have an inverse model which, due to the simple equations of the model, can analytically compute the required open-loop torque to achieve a desired joint trajectory. The model proposed by Bhanpuri et al. (2014) compensated for the static perturbations with a long-latency (100 ms) negative feedback control on the error between desired (minimum-jerk) and actual elbow joint angle trajectory. The model proposed by Kalveram and Seyfarth (2009) is quite similar. However, it proposes zero-time-delay negative feedback representing the tunable mechanical elasticity of the muscles. Both models did not consider muscle contraction dynamics and, therefore, do not allow to investigate the sensorimotor interplay in consequence of such perturbations. The model presented here transfers these control concepts to the more physiologically detailed musculoskeletal model. As a consequence, it validly reproduces the response to both static and dynamic perturbations and, in addition, allows for further insights into the neuromuscular interplay of arm movements and internal dynamics in response to such perturbations (Figure 9), as we will discuss in the following.

### 4.1. Unperturbed Movements: Intermittent Open-Loop Control

In our model, the unperturbed reference movement is solely generated by an open-loop command. Although other musculoskeletal models show that feedback signals may play a role in the generation of unperturbed arm movements (e.g., Bizzi et al., 1992; Desmurget and Grafton, 2000; Kistemaker et al., 2006; Kambara et al., 2009), we chose this approach to closely resemble the motor control models previously used to investigate these perturbations (Kalveram et al., 2005; Bhanpuri et al., 2014). To be able to determine an open-loop control signal in our neuro-muscular model, we parametrized the control signal resulting in a piecewise constant stimulation over time for each muscle (Figure 3). Hereby we exploit the advantage of neuro-musculoskeletal models that allow stable open-loop starting and target positions due to the passive visco-elastic characteristics of the muscles and the length dependence of the activation dynamics (Kistemaker et al., 2005, 2007). Such so-called *equilibrium points* (Feldman, 1986) can be found without and with gravity. Previously, complete equilibrium trajectories have been proposed as control concept for smooth movements, where each point on the kinematic trajectory is an equilibrium point (Flash and Hogan, 1985; Bizzi et al., 1992). Kistemaker et al. (2006) composed their open-loop signal from several intermittent equilibrium points resulting in a piecewise constant stimulation over time for every muscle. Also, our controller generates an intermittent purely open-loop stimulation to generate the desired movement.

This intermittent control has two characteristics worth mentioning. Firstly, it is interesting to see that it actually results in a smooth movement—without gravity (Figure 6A) and with gravity (Figure 8A). This is a result of the activation dynamics, the visco-elastic properties of the muscle-tendon units, and the inertia of the lower arm. Secondly, it can achieve the required velocity purely controlled by an open-loop signal. This is in contrast to previous intermittent equilibrium point control, where proprioceptive feedback was included to achieve fast movements (Kistemaker et al., 2006). While their intermittent control points all were equilibrium points taken directly from their desired trajectory, the intermittent control parameters in our optimization are free, allowing us to match the velocity of the experiments purely by open-loop control.

### 4.2. Perturbed Movements: Hierarchical Levels of Feedback

An external force applied to the arm during the movement generates a deviation from the planned/anticipated movement. With our model, we can study the response of the neuro-musculoskeletal system on several hierarchical levels.

#### 4.2.1. Musculoskeletal Response

The evaluation of the stimulation signals (Figure 9) shows that the relative contribution of the feedback signal is small (always <16% for 25 ms delay, <34% for 50 ms delay, even less for the static perturbations), i.e., the stimulation comes predominantly from the open-loop controller. We therefore repeated the perturbation simulations with open-loop control. Interestingly, even when solely driven by an open-loop command, the system already shows a similar response to the perturbations as the healthy subjects in the experiments (Figures 6A, 8A). The reason for this is that the antagonistically arranged muscle models account for the non-linear force-length-velocity relationship of muscle fibers and the passive non-linear elasticities of tendons. This relationship basically acts as a zero-time-delay peripheral feedback (previously termed *preflex*, Brown et al., 1995). In consequence, the force produced by the muscles changes not only with changes in stimulation but also with changes in the length and contraction velocity of the muscle fibers—which change during the movement. Hence, our open-loop controlled system includes an internal feedback mechanism on the muscular level. The role of this effect becomes strikingly clear in comparison to a torque-based model that was able to reproduce the unperturbed movement but failed to adequately respond to the perturbations in the open-loop scenario. So, the difference between the open-loop controlled musculoskeletal model (Figures 6A, 8A) and the torque-driven model (Figures 6C, 8C) is the consequence of the immediate physical response due to the impedance of the muscular system. The relevance of this immediate response is also emphasized by the velocity quotient evaluated at 37.5 ms after the perturbation (Figure 7A) as it is independent of the feedback signal and thus reflects the musculoskeletal response. The resemblance of this velocity quotient to the experiment indicates that the system's state is adequately represented as it characterizes the initial response to perturbations. This means that the first zero-time-delay response is provided by the muscle-tendon units and it shows already correct qualitative responses to the perturbations. This indicates that the relative importance of feedback over feed-forward may be diminishing in the presence of muscular characteristics (Pinter et al., 2012), which is particularly interesting with respect to assistive devices for rehabilitation. Furthermore, the capability of the musculoskeletal system to stabilize against external perturbations (Brown et al., 1996; Wagner et al., 2007) may allow reducing the informational control effort (Haeufle et al., 2014a, 2020) by exploiting the capability of morphological computation of the biomechanical system (Ghazi-Zahedi et al., 2016).

#### 4.2.2. First Neuronal Response

The next level of response to the perturbation is the short- or long-latency feedback mechanism that we implemented in our model. Both the short- and the long-latency feedback lead to the same qualitative behavior (see Supplementary Material for short-latency results). Depending on the type of perturbation, the feedback in our model helps to bring the simulated trajectory closer to the experiment (Table 1). For the damping perturbations, the closed-loop controlled system is less sensitive to the perturbations than the version without feedback, because the feedback works against the perturbations during the whole movement. Therefore, with feedback, the movement is closer to the unperturbed trajectory which is closer to the experiment than the open-loop version of the model. When perturbing the inertia properties, feedback enhances the effect of the perturbation which leads to a trajectory that is further away from the experiment. This becomes visible in the quantification criterion *dysmetria*, which evaluates the deviation in the target position due to the static perturbations. On the other hand, the quantification criterion *early velocity* for the static perturbations is only little affected by the feedback because it is measured in the early phase of the movement where feedback does not play a big role due to its delay. Also for the dynamic perturbation, feedback improves the response. However, this is only little reflected in the chosen quantification criterion (velocity quotient, Figure 7A) since it takes into account the velocity before the perturbation and 37.5 ms or 100 ms after the perturbation, respectively, while the feedback delay is 50 ms. Hence, the model prediction benefits from the sensorimotor integration on the lower-level reflex level in response to these perturbations.

#### 4.2.3. More Complex Long-Latency Feedback and Higher-Level Adaptation

In addition to the musculoskeletal response and the simple short- and long-latency feedback, more complex long-latency feedback and higher-level control would be able to further handle the late consequences of perturbations. While data on dynamic perturbations in human arm movements indicate only a small response in the time-window of short-latency reflexes—as in our model—it shows well-tuned and adequate responses of long-latency reflexes (45 ms to 100 ms, Kurtzer et al., 2008). Such long-latency feedback (100 ms) has been used by Bhanpuri et al. (2014) to compensate for the static perturbations in their torque-driven model, an effect we can reproduce in our torque model as well (Figures 6D, 8D) where responses get closer to the experiment than without feedback but tend to oscillate around the final positions. Currently, our neuro-musculoskeletal model does only consider the muscle-fiber-length- and velocity-dependent aspects of long-latency reflexes. More complex or higher-level feedback strategies seem not necessary to reproduce the immediate perturbation response.

#### 4.2.4. Relevance for Motor Control

We interpret these findings such that muscles generate an immediate zero-time-delay impedance response. Short-latency feedback and our simplified representation of long-latency feedback have little influence, and not necessarily beneficial for all types of unexpected interaction forces. More complex long-latency feedback could then consider an internal model of limb dynamics (Kurtzer et al., 2008, 2014) for an adequate complex response. However, this is not implemented in our model (4). Therefore, the detailed modeling of the low-level neuro-muscular control mechanism is suggested to be important to understand (i) higher-level control mechanisms, (ii) their disturbances in patients with movement disorders and (iii) to develop effective assistive devices to compensate for those disturbances.

### 4.3. Model Assumptions and Limitations

To derive control parameters, we made a few assumptions. The most prominent assumption was the triphasic pattern (2) which was our approach to tackle the inverse model problem: finding required control signals for the desired trajectory. Our approach was inspired by the observation of triphasic patterns in muscle surface electromyograms (EMG) (see e.g., Wachholder and Altenburger, 1926; Wierzbicka et al., 1986; Kistemaker et al., 2006) and has been discussed in detail above (4.1). Other approaches tackled this inverse problem by reducing the biomechanical complexity: Examples are ideal torque generators in the joints (e.g., Bhanpuri et al., 2014), linear or non-linear spring, and spring-damper models (e.g., Kalveram et al., 2005; Kalveram and Seyfarth, 2009), or simplified muscle models which contain no tendons, no activation dynamics and an entire model without any neuronal delays (Teka et al., 2017). Furthermore, inverse relations between a desired movement and control may also be resolved for musculoskeletal models by more elaborate optimizations (Todorov, 2004; Kistemaker et al., 2014; Driess et al., 2018), although it is not easy to determine a physiologically relevant cost function (Todorov, 2004; Berret et al., 2011; Loeb, 2012). A third option entirely circumvents the inverse problem by iterative motor learning (e.g., Gribble and Ostry, 2000; Kambara et al., 2009).

Some of the control parameters were chosen by hand while others were optimized to match the unperturbed or perturbed trajectories (see Table 2 in the Appendix). To investigate the influence of the control parameters on the resulting movement, we performed a sensitivity analysis (see Appendix). We quantified the sensitivity to small changes of the control parameters in two ways: (a) by measuring the effect on the trajectory (time-based measure) or (b) by measuring the effect on a scalar characteristic measure that describes the behavior [cost function used in the optimization (5); velocity quotient (6)]. Note that these sensitivity indicators need to be treated carefully as for example the relative sensitivity to a change of the time delay δ around the reference value of 50 ms is relatively high (Figure 11 in the Appendix) while a change of the time delay from 50 ms to 25 ms or 100 ms without re-optimizing the other control parameters has only little influence on the qualitative behavior in reaction to the perturbations (results not shown here). This is due to the fact that the chosen state variables are sums over several cases and non-linear functions of the input parameters. The influence is even smaller when re-optimizing the other control parameters after changing the time delay from 50 ms to 25 ms (see Supplementary Material) or 100 ms (not shown here). In doing so, the changes in the delay can be compensated for by adapting the other control parameters. We assume that the nervous system similarly adapts the motor control when for example the feedback delay changes. Overall, the sensitivity analysis shows that some control parameters do have a relevant influence on the results. However, the overall behavior is only little affected when the other control parameters are re-optimized to compensate for the change.

The second assumption for the control is further related to the biomechanical representation: the type of feedback. Torque models and other simplified models often use the joint angle as the control level to account for deviations between desired and actual trajectory (e.g., Kalveram et al., 2005; Bhanpuri et al., 2014). In our model, however, we use muscle spindle signal based feedback and assume that it provides direct feedback of the muscle fiber length and contraction velocity. We neglect other types of proprioceptive feedback, for example from Golgi tendon organs, which may provide a link to joint-based control (Kistemaker et al., 2013). Furthermore, more detailed representations of the proprioceptors (Loeb and Mileusnic, 2015) allow for a detailed analysis of, e.g., the role of alpha-gamma co-activation (Lan and Zhu, 2007; Lan and He, 2012).

Finally, one crucial assumption is the neuronal delay, as it strongly influences the interpretation of the location of the feedback in the neuronal hierarchy. By assuming zero time delay, Kalveram et al. (2005) located the negative feedback control at the biomechanical level—a common approach which is not always clearly separated from afferent signals (e.g., Teka et al., 2017). Experimental findings show that the short-latency reflex can produce more sophisticated responses to perturbations than previously thought (Weiler et al., 2019). This short-latency feedback occurs after a time delay of ~20 ms to 50 ms after a perturbation (Shemmell et al., 2010; Pruszynski and Scott, 2012; Kurtzer et al., 2014). Other delays in the order of 50 ms to 100 ms represent long-latency reflexes (Shemmell et al., 2010; Pruszynski and Scott, 2012; Kurtzer, 2015; Weiler et al., 2016), as used for example by Gribble and Ostry (2000), Bhanpuri et al. (2014). Several studies have shown that the long-latency stretch response plays an important role in the reaction to mechanical perturbations in goal-directed reaching movements (e.g., Kurtzer et al., 2014; Weiler et al., 2016). In our model, using 25 ms delay, the implemented feedback mechanism represents a simplified model of the spinal, mono-synaptic muscle spindle reflex (Pruszynski and Scott, 2012; Weiler et al., 2019), assuming that the muscle spindles provide accurate time-delayed information about the muscle fiber lengths and contraction velocities (Kistemaker et al., 2006). However, this model of the afferent feedback does not accurately reflect the natural muscle spindle feedback which is only sensitive to the muscle's local stretch (Kurtzer et al., 2014, see Scott, 2016 for an overview) while our formulation reacts to stretch and shortening. Therefore, choosing a time delay of 50 ms and thus modeling a long-latency reflex seems more appropriate. However, this model considers only the muscle-fiber-length- and contraction-velocity-dependent part of the long-latency feedback and neglects other aspects. This becomes visible in the velocity quotient after 100 ms (Figure 7A) which characterizes the behavior at the end of the first neuronal response. It is further away from the experiments than the velocity quotient after 37.5 ms, suggesting that our long-latency feedback model does not include all relevant feedback mechanisms. Experimental findings indicate that long-latency feedback represents the net impact of spinal and cortical circuits and thus includes several independent processes (e.g., Pruszynski et al., 2011; Kurtzer et al., 2014) that for example account for limb biomechanics (Kurtzer, 2015) or evoke responses in muscles that were not stretched (Weiler et al., 2018). The reaction after more than 100 ms after the perturbation is influenced by more complex and higher-level feedback mechanisms and voluntary activities (Pruszynski and Scott, 2012; Kurtzer, 2015; Weiler et al., 2016) that are not represented in our model. Although the resulting reactions of our model to the perturbations seem quite sensitive to the chosen delay time (see Sensitivity Analysis in the Appendix), the results were quite similar for choosing 25, 50, or even 100 ms delay (the latter results are not shown in this contribution). Our model reproduces the response to the perturbation by using short-latency feedback (25 ms) which represents spinal control layers or long-latency feedback (50 ms) which has spinal and supraspinal influences. Once more this emphasizes the decentralized control. However, as the feedback contribution was rather small and did not improve the response in all cases, it is likely that more sophisticated models, which may, for example, include multiple layers of feedback including more complex long-latency feedback (Kurtzer et al., 2008) would improve the model prediction.

As with the control and feedback assumptions, also the level of detail of the musculoskeletal model has its limitations. Although our muscle model represents contraction dynamics quite well (Haeufle et al., 2014b), it does not consider recent findings on the behavior of muscles under eccentric loading conditions (Tomalka et al., 2017), on the possible role of short-range stiffness (Nichols and Houk, 1976; De Groote et al., 2017), or the effect of transversal loading (Siebert et al., 2014). As we see significant force changes in the dynamic perturbations originating from the muscle's passive characteristics (Figure 9), these new findings may also influence the response. Ultimately, for the study of internal contact forces, finite-element models may allow a more detailed analysis (Röhrle et al., 2016) but significantly increase the complexity of finding an adequate controller (Martynenko et al., 2017).

### 4.4. Conclusion

For our study, the focus was on the valid prediction of the response to static and dynamic external perturbations while providing the possibility to investigate the neuromuscular interplay at a level that allows predicting muscle-bone contact forces and joint loadings. As our model with its assumptions and limitations still fulfills the initially stated criteria, we consider it a starting point to further develop models with the integrated use: studying motor control and ergonomics with the same model for research questions where they overlap, e.g., for the development and ergonomic risk assessment of assistive devices.

## Data Availability Statement

The datasets generated for this study are available on request to the corresponding author.

## Author Contributions

KS, WI, and DH: project concept and manuscript. KS and DH: numerical experiments.

## Conflict of Interest

The authors declare that the research was conducted in the absence of any commercial or financial relationships that could be construed as a potential conflict of interest.
