# Supplementary material for: Predicting Perturbed Human Arm Movements in a Neuro-Musculoskeletal Model to Investigate the Muscular Force Response
Source: Front Bioeng Biotechnol. 2020 Apr 21;8:308. doi: 10.3389/fbioe.2020.00308 (PMC7186382; doi:10.3389/fbioe.2020.00308)
Supplement: Supplementary file 1 [file Data_Sheet_1.PDF]

Appendix to "Predicting perturbed human arm movements in a neuro-musculoskeletal model to investigate the muscular force response"

Katrin Stollenmaier, Winfried Ilg, Daniel F.B. Haeufle
Department of Cognitive Neurology, Hertie Institute for Clinical Brain Research and
Werner Reichardt Centre for Integrative Neuroscience, University of Tübingen, Tübingen, Germany
Correspondence: Katrin Stollenmaier: katrin.stollenmaier@uni-tuebingen.de

			① without external torque	② with external torque, flexion	② with external torque, extension
initial position	$u^{\text{des.},0}$		0^*	0^*	0^*
	u_i^0	Elbow flexor	$0.0100^{\#}$	$0.0704^{\#}$	$0.198^{\#}$
		Elbow extensor	$0.0295^{\#}$	$0.0101^{\#}$	$0.0165^{\#}$
		biart. flexor	$0.0101^{\#}$	$0.0101^{\#}$	$0.0539^{\#}$
		biart. extensor	$0.0101^{\#}$	$0.0101^{\#}$	$0.0286^{\#}$
acceleration	$u^{\text{min.}}$		0.005^*	0.005^*	0.005^*
	$u^{\text{acc.}}$		$0.116^{\triangle}$	$0.140^{\triangle}$	$4.46 \times 10^{-4\triangle}$
	t_1		$0.410\,\text{s}^{\triangle}$	$0.509\,\text{s}^{\triangle}$	$0.382\,\text{s}^{\triangle}$
deceleration	$u^{\text{des.,dec.}}$		$0.240^{\diamond}$	$0.240^{\diamond}$	$0.240^{\diamond}$
	$u_i^{\text{dec.}}$	Elbow flexor	$0.267^{\#}$	$0.267^{\#}$	$0.204^{\#}$
		Elbow extensor	$0.0898^{\#}$	$0.0898^{\#}$	$0.259^{\#}$
		biart. flexor	$0.261^{\#}$	$0.261^{\#}$	$0.221^{\#}$
		biart. extensor	$0.229^{\#}$	$0.229^{\#}$	$0.243^{\#}$
	t_2		$0.7\,\text{s}^*$	$0.7\,\text{s}^*$	$0.7\,\text{s}^*$
final position	$u^{\text{des.,final}}$		0.3^*	0.3^*	0.3^*
	u_i^{final}	Elbow flexor	$0.330^{\#}$	$0.334^{\#}$	$0.221^{\#}$
		Elbow extensor	$0.104^{\#}$	$0.0928^{\#}$	$0.325^{\#}$
		biart. flexor	$0.324^{\#}$	$0.326^{\#}$	$0.266^{\#}$
		biart. extensor	$0.284^{\#}$	$0.283^{\#}$	$0.307^{\#}$
	k_p		$0.2^{\diamond}$	$0.2^{\diamond}$	$0.2^{\diamond}$
	k_d		$0.15^{\diamond}$	$0.15^{\diamond}$	$0.15^{\diamond}$
	δ		0.05^*	0.05^*	0.05^*

Table 2. Control parameters used in the computer simulation of external perturbations during point-to-point movements in a horizontal plane. For a better understanding of the abbreviations see Figure 3 in the main part of the manuscript. Gray values indicate that the same values as for ① have been used. Meaning of the symbols: $*$: quantities that we set to a fixed value, #: optimized such that there is an equilibrium point at this position given the desired level of co-contraction, $\triangle$: optimized to match the unperturbed trajectory for case ①, $\diamond$: optimized to match the perturbed trajectories for case ①.

5 SENSITIVITY ANALYSIS

We perform a local, first order differential sensitivity analysis by varying the control parameters listed in Table 2 one-at-a-time (e.g., Dickinson and Gelinas, 1976; Rockenfeller, 2016; Morio, 2011). We calculate absolute and relative sensitivities as defined below and choose scalar state variables for the different cases. The relative change of a parameter x is set to $\Delta = 1 \times 10^{-3} \cdot x$, i.e. we perform a local sensitivity analysis without taking into account the physiological range of the parameters (Rockenfeller et al., 2015; ten Broeke, 2017).

5.1 Absolute sensitivity of the trajectory per time step

We define the absolute sensitivity coefficient as

$$S_{\text{abs.}} := \frac{f(x+\Delta) - f(x)}{\Delta} \,, \tag{S1}$$

which approximates the derivative of $f(x)$ in the direction of the parameter x for Δ small enough.

As scalar state variable we choose the difference between the simulated and the experimental trajectory. For every time step, we sum this trajectory difference over all four perturbation cases and add the unperturbed case for each the static and the dynamic perturbations. In doing so, the time evolution of the sensitivity of the trajectory to the control parameters can be investigated.

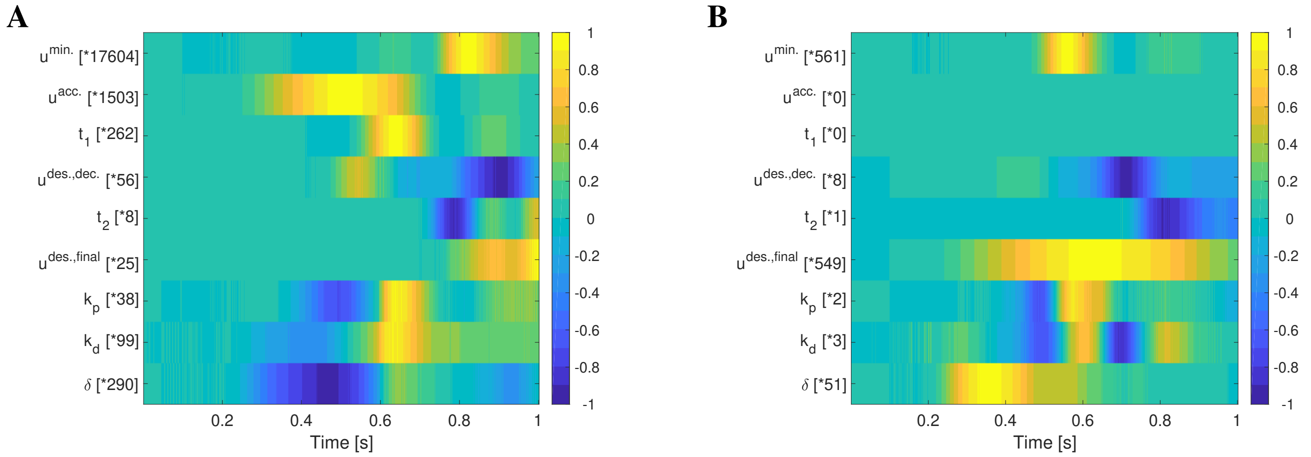

Figure 10: Normalized absolute sensitivity $S_{\text{abs.}}$ over time for **A** the static and **B** the dynamic perturbations. Each line represents one of the varied parameters. For visualization purposes, all sensitivities are normalized by dividing them by the maximum absolute value per parameter. This maximum absolute value is shown in the square brackets after the name of each parameter to denote the differences in magnitudes.

5.2 Relative sensitivity of one characteristic measure

We define the relative sensitivity coefficient as

$$S_{\text{rel.}} := \frac{\frac{f(x+\Delta)-f(x)}{f(x)}}{\frac{\Delta}{x}} = S_{\text{abs.}} \cdot \frac{x}{f(x)} \,, \tag{S2}$$

i.e. it is a normalized approximation for the derivative of the state variable $f(x)$ in the direction of the parameter x for Δ small enough (e.g., Lehman and Stark, 1982). This relative sensitivity is sometimes referred to as *elasticity* (ten Broeke, 2017). The advantage of the normalization is that the resulting

sensitivity indicator is easier to interpret and more comparable between different cases or even across models (Scovil and Ronsky, 2006; Rockenfeller et al., 2015). Using this definition, the relative sensitivity indicates the percentage change in the state variable per percentage change in the parameter value. For example, a relative sensitivity $S_{\text{rel.}} = 2$ indicates that a $m\%$ change of the input parameter x results in a $2m\%$ change of the output $f(x)$.

As scalar state variable that describes the behavior in reaction to the perturbations, we choose a measure that describes the characteristics of the reaction to the perturbations. For the static perturbations, we choose the cost function (Equation 5 in the main part of the manuscript). For the dynamic perturbations, we calculate the difference between the velocity quotient (Equation 6 in the main part of the manuscript) in the simulation and the one in the experiment and sum it over all four types of perturbations.

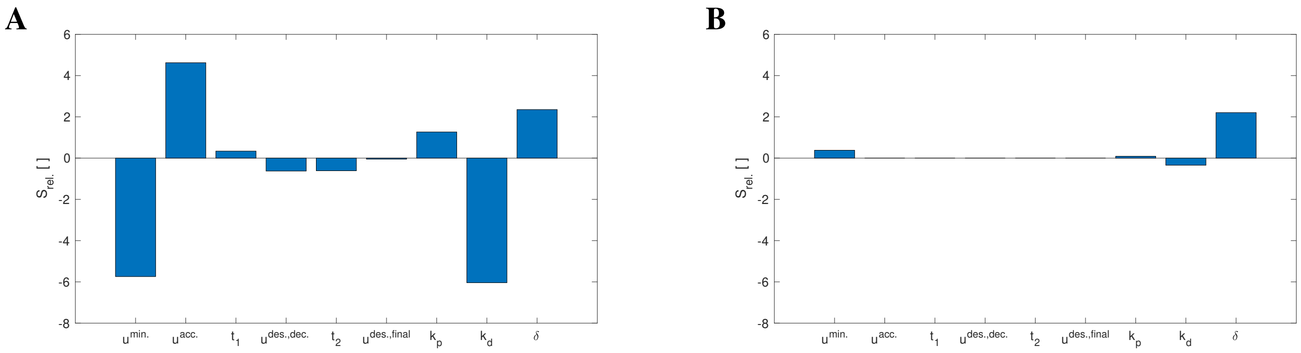

Figure 11: Relative sensitivity $S_{\text{rel.}}$ for each of the varied parameters for **A** the static perturbations using the cost function (Equation 5 in the main part of the manuscript) as characteristic measure and **B** the dynamic perturbations using the summed difference between the velocity quotient (Equation 6 in the main part of the manuscript) in the simulation and in the experiment.

REFERENCES

Dickinson, R. P. and Gelinas, R. J. (1976). *Sensitivity Analysis of Ordinary Differential Equation Systems - A Direct Method*. Tech. rep.

Lehman, S. and Stark, L. (1982). Three Algorithms for Interpreting Differential Equations: Sensitivity. *Mathematical Biosciences* 62, 107–122

Morio, J. (2011). Global and local sensitivity analysis methods for a physical system. *European Journal of Physics* 32, 1577–1583

Rockenfeller, R. (2016). *On the Application of Mathematical Methods in Hill – Type Muscle Modeling : Stability , Sensitivity and Optimal Control by*. Ph.D. thesis

Rockenfeller, R., Günther, M., Schmitt, S., and Götz, T. (2015). Comparative Sensitivity Analysis of Muscle Activation Dynamics. *Computational and Mathematical Methods in Medicine* 2015, 1–16

Scovil, C. Y. and Ronsky, J. L. (2006). Sensitivity of a Hill-based muscle model to perturbations in model parameters. *Journal of Biomechanics* 39, 2055–2063

ten Broeke, G. (2017). *Sensitivity analysis methodologies for analysing emergence using agent-based models*. Ph.D. thesis
