# Supplementary material for: Predicting Perturbed Human Arm Movements in a Neuro-Musculoskeletal Model to Investigate the Muscular Force Response
Source: Front Bioeng Biotechnol. 2020 Apr 21;8:308. doi: 10.3389/fbioe.2020.00308 (PMC7186382; doi:10.3389/fbioe.2020.00308)
Supplement: Supplementary file 2 [file Data_Sheet_2.pdf]

Supplementary material describing Arm26

Katrin Stollenmaier and Daniel F.B. Haeufle

Department of Cognitive Neurology, Hertie Institute for Clinical Brain Research and

Werner Reichardt Centre for Integrative Neuroscience, University of Tübingen, Tübingen, Germany

Correspondence: Katrin Stollenmaier: katrin.stollenmaier@uni-tuebingen.de

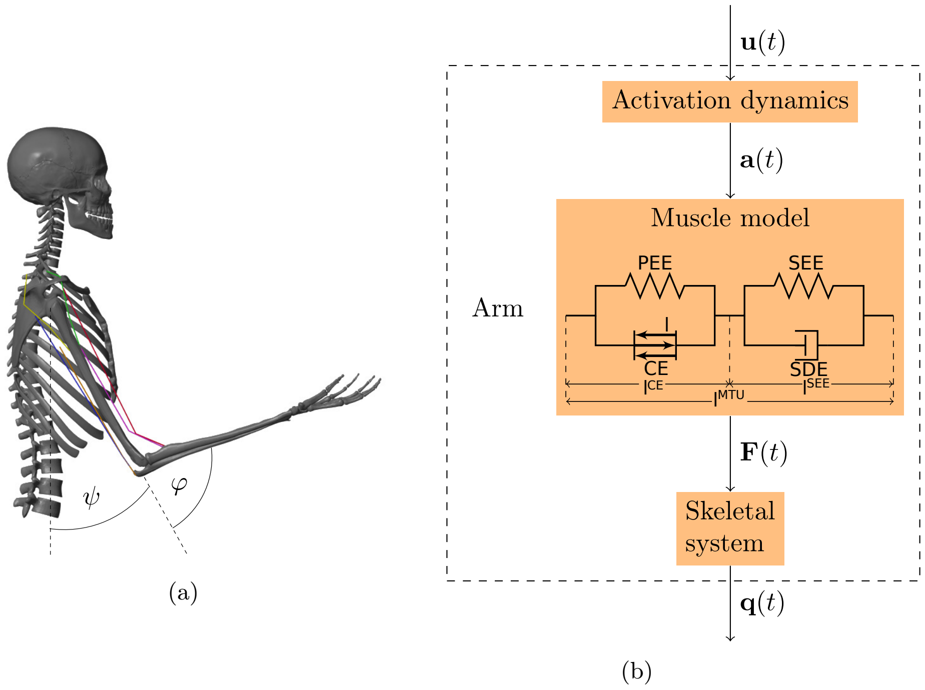

Figure 1: (a) Visualization of the musculoskeletal model of the arm and the definition of the shoulder angle $\psi(t)$ and the elbow angle $\varphi(t)$ and (b) Structure of the arm model: the motor command $\mathbf{u}(t)$ is fed into the model of the activation dynamics of muscles which relates the neuronal stimulation to muscular activity $\mathbf{a}(t)$ that drives the muscle model. The muscles produce forces $\mathbf{F}(t)$ that act on the skeletal system resulting in a simulated movement $\mathbf{q}(t) = [\varphi(t), \psi(t)]$ of the arm.

The neuro-musculoskeletal model *Arm26* consists of a musculoskeletal model of the arm with two degrees of freedom actuated by six muscles and a controller. The model is implemented using Matlab® R2018a/Simulink® with the Simscape Multibody™environment. For a better overview, the implementation of the model is divided into three parts: the mechanical part (representing the bone structure and the muscle routing), the actuation of this mechanical part (muscle-tendon structures) and the controller (nervous system) which provides the input to the actuation part.

1 Musculoskeletal model of the arm: Mechanics and Actuation

The musculoskeletal model *Arm26* of the human arm uses the same geometry and muscle parameters as the simulation model described in Driess et al. (2018) which is based on Bayer et al. (2017). It consists of two rigid bodies (lower and upper arm) that are connected via two one-degree-of-freedom revolute joints that represent the shoulder and elbow joint. This multibody system is actuated by six muscle-tendon units (MTU), four monoarticular and two biarticular muscles (see Figure 1a). The muscles are modeled as lumped muscles, i.e. they represent a multitude of anatomical muscles:

1. Monarticular Elbow Flexor (MEF) (short: Elbow Flexor (EF)):
   m. brachioradialis, m. brachialis, m. pronator teres, m. extensor carpi radialis
2. Monarticular Elbow Extensor (MEE) (short: Elbow Extensor (EE)):
   m. triceps lateralis, m.triceps medialis, m. an-coneus, m. extensor carpi ulnaris
3. Biarticular Elbow Flexor Shoulder Anteversion (BEFSA) (short: Biarticular Flexor (BF)):
   m. biceps brachii caput longum and caput breve
4. Biarticular Elbow Extensor Shoulder Retroversion (BEESR) (short: Biarticular Extensor (BE)):
   m. triceps brachii caput longum
5. Monoarticular Shoulder Anteversion (MSA) (short: Shoulder Flexor (SF)):
   m. deltoideus (pars clavicularis, anterior, lateral), m. superior pectoralis major, m. coracobrachialis
6. Monoarticular Shoulder Retroversion (MSR) (short: Shoulder Extensor (SE)):
   m. deltoideus (pars spinalis, posterior), m. latissimus dorsi

The MTU structure is modeled using an extended Hill-type muscle model as described in Haeufle et al. (2014) with muscle activation dynamics as introduced by Hatze (1977). The muscle model is a macroscopic model consisting of four elements: the contractile element (CE), the parallel elastic element (PEE) and the serial elastic element (SEE) and serial damping element (SDE), as illustrated in Figure 1b. The inputs to the muscle model are the length of the MTU l^{MTU}, the contraction velocity of the MTU $\dot{l}^{\mathrm{MTU}}$ and the muscular activity a. The output of the muscle model is a one-dimensional muscle force F^{MTU}. This force drives the movement of the skeletal system. For the routing of the muscle path around the joints, deflection ellipses are implemented as described by Hammer et al. (2019) (see Figure 2). The muscle path can move within these ellipses and is deflected as soon as it touches the boundary.

All in all, the governing model dependencies for all muscles $i = 1, ..., n$ are:

$$\begin{aligned}
\dot{l}_i^{\mathrm{CE}} &= f_{\mathrm{CE}}(l_i^{\mathrm{CE}}, l_i^{\mathrm{MTU}}, \dot{l}_i^{\mathrm{MTU}}, a_i) && (1)\\
\dot{a}_i &= f_a(a_i, u_i, l_i^{\mathrm{CE}}) && (2)\\
F_i^{\mathrm{MTU}} &= f_F(l_i^{\mathrm{MTU}}, \dot{l}_i^{\mathrm{MTU}}, l_i^{\mathrm{CE}}, a_i) && (3)\\
\ddot{\mathbf{q}} &= f_q(\dot{\mathbf{q}}, \mathbf{q}, \mathbf{F}^{\mathrm{MTU}})\,, && (4)
\end{aligned}$$

where $\mathbf{q}$ denotes a generalized state vector, in this case it can be defined as $\mathbf{q} = [\varphi, \psi]$ and $\mathbf{F}^{\mathrm{MTU}} = \{F_i^{\mathrm{MTU}}\}_{i=1}^n$.

The mechanical parameters of the arm segments are taken from Kistemaker et al. (2006) and can be found in Table 1. The positions and sizes of the deflection ellipses were chosen in order to match moment arms in literature (see Figure 3) and can be found in Listing 1. For more details on this see Suissa (2017). The (non-)muscle-specific parameters can be found in Table 2 and Table 3.

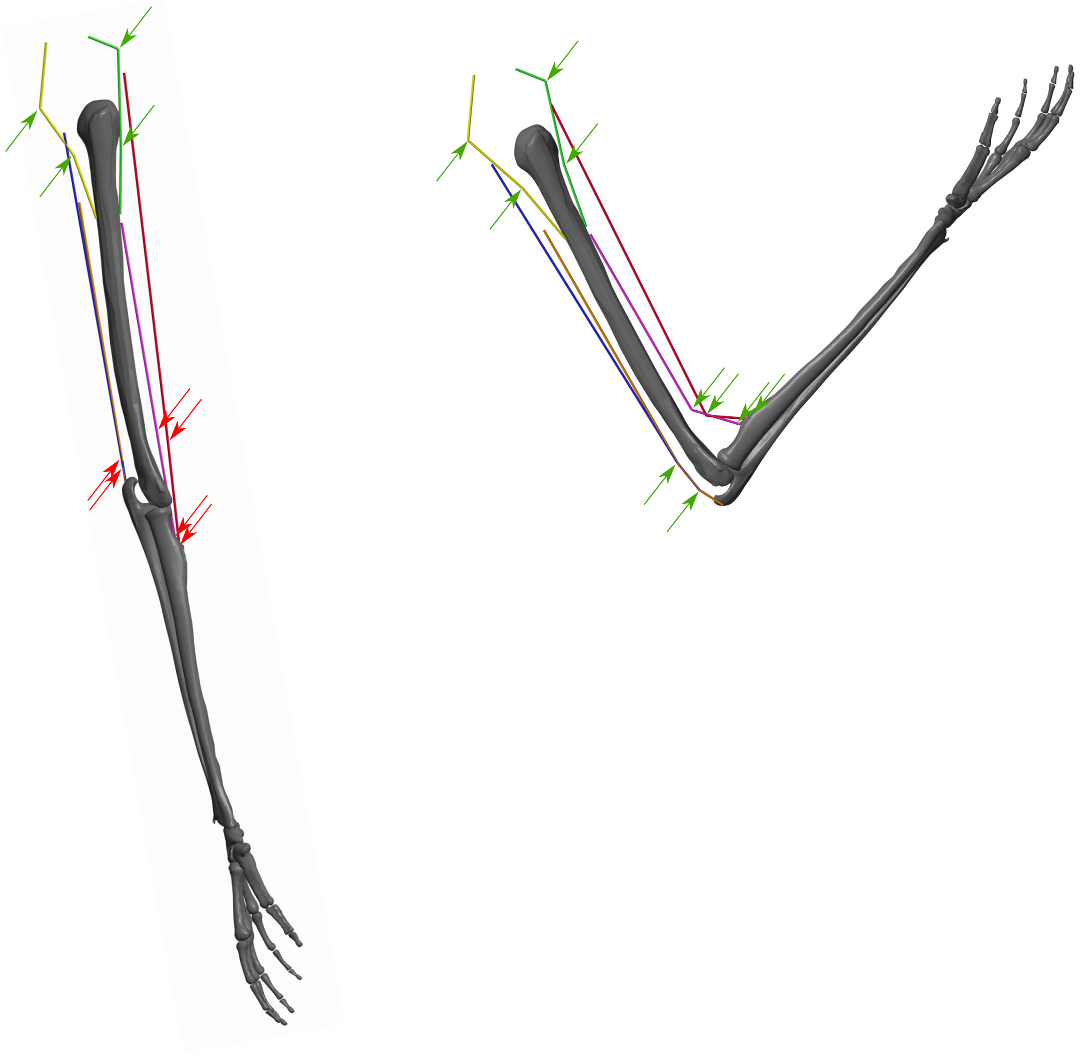

Figure 2: Illustration of the positions of the deflection ellipses that are used for the muscle routing in two different arm positions. Green arrows indicate active ellipses that deflect the muscle path, while red arrows indicate inactive ellipses that do not change the muscle path.

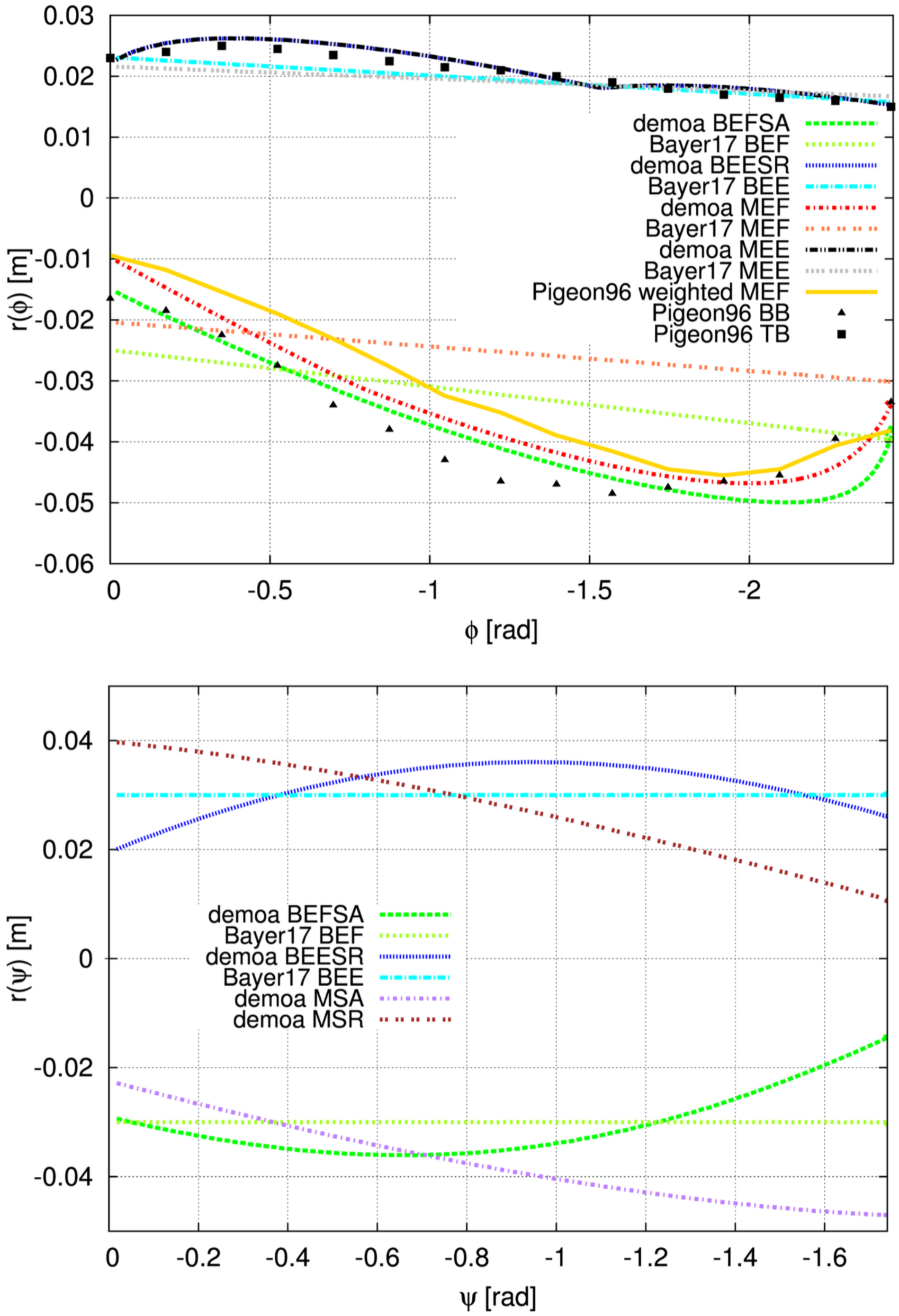

Figure 3: Comparison of the moment arms of the muscles in the model with simulation and experimental data from literature for the elbow muscles (upper plot) and the shoulder muscles (lower plot). The lines marked with "demoa" refer to the model by Suissa (2017) on which our model is based (for the naming of the muscles see Table 2). We use the same geometry and position and size of the ellipses, so our moment arms are the same as in the "demoa" model. The moment arms are compared to a calculatory model by Bayer et al. (2017) (here M/B stands for mono- and biarticular, E stands for elbow and F/E stands for flexion and extension, respectively) and to experimental data. The black marks show experimental data of the biceps brachii (BB) and the triceps brachii (TB) taken from Pigeon et al. (1996). The yellow line shoes a weighted combination of the monoarticular flexor muscles that are represented by the MEF in the model. They are weighted according to their proportion of the joint torques, see Sobotta (2010); Aumüller et al. (2017). The figure was taken from Suissa (2017) with kind permission of the author.

	Length [m]	d [m]	Mass [kg]	I [kgm^2]	I with exoskeleton [kgms^2]
Upper arm	0.335	0.146	2.10	0.024	0.024
Lower arm	0.263	0.179	1.65	0.025	0.1118

Table 1: Mechanical parameters of the skeletal structure (Kistemaker et al. (2006)) with d=distance from proximal joint to center of mass and I=moment of inertia with respect to the center of mass. Last column: when comparing to experiments, the inertia properties of the lower arm can be adapted according to an arm that is attached to an exoskeleton robot that was used by Bhanpuri et al. (2014).

	$F^{\max}$ [N]	$l^{\mathrm{CE,opt}}$ [m]	$l^{\mathrm{SEE,0}}$ [m]
Monarticular Elbow Flexor (MEF)	1420	0.092	0.182
Monarticular Elbow Extensor (MEE)	1550	0.093	0.187
Monoarticular Shoulder Anteversion (MSA)	838	0.134	0.039
Monoarticular Shoulder Retroversion (MSR)	1207	0.140	0.066
Biarticular Elbow Flexor Shoulder Anteversion (BEFSA)	414	0.151	0.245
Biarticular Elbow Extensor Shoulder Retroversion (BEESR)	603	0.152	0.260

Table 2: Muscle-specific actuation parameters (Kistemaker et al. (2006) and Kistemaker et al. (2013)), with $F^{\max}$: maximum isometric force, $l^{\mathrm{CE,opt}}$: optimal length of the contractile element, $l^{\mathrm{SEE,0}}$ rest length of the serial elastic element. The lengths of $l^{\mathrm{CE,opt}}$ and $l^{\mathrm{SEE,0}}$ were adapted to match the muscle path routed through the ellipses in order to allow for a big range of motion. For this parameter adaptation method see Suissa (2017).

	Parameter	Unit	Value	Source	Description
CE	ΔW^{des}	[ ]	0.45	similar to Bayer et al. (2017); Kistemaker et al. (2006)	width of normalized bell curve in descending branch, adapted to match observed force-length curves
	ΔW^{asc}	[ ]	0.45	similar to Bayer et al. (2017); Kistemaker et al. (2006)	width of normalized bell curve in ascending branch, adapted to match observed force-length curve
	$\nu^{\text{CE,des}}$	[ ]	1.5	Mörl et al. (2012)	exponent for descending branch
	$\nu^{\text{CE,asc}}$	[ ]	3.0	Mörl et al. (2012)	exponent for ascending branch
	$A^{\text{rel},0}$	[ ]	0.2	Günther (1997)	parameter for contraction dynamics: maximum value of A^{rel}
	$B^{\text{rel},0}$	[1/s]	2.0	Günther (1997)	parameter for contraction dynamics: maximum value of B^{rel}
	$\mathcal{S}^{\text{ecc}}$	[ ]	2.0	van Soest et al. (1993)	relation between $F(v)$ slopes at $v^{\text{CE}} = 0$
	$\mathcal{F}^{\text{ecc}}$	[ ]	1.5	van Soest et al. (1993)	factor by which the force can exceed F^{isom} for large eccentric velocities
PEE	$\mathcal{L}^{\text{PEE},0}$	[ ]	0.95	Günther (1997)	rest length of PEE normalized to optimal length of CE
	ν^{PEE}	[ ]	2.5	Mörl et al. (2012)	exponent of F^{PEE}
	$\mathcal{F}^{\text{PEE}}$	[ ]	2.0	Mörl et al. (2012)	force of PEE if l^{CE} is stretched to ΔW^{des}
SDE	D^{SDE}	[ ]	0.3	Mörl et al. (2012)	dimensionless factor to scale $d^{\text{SDE,max}}$
	R^{SDE}	[ ]	0.01	Mörl et al. (2012)	minimum value of d^{SDE} (at $F^{MTU} = 0$), normalized to $d^{\text{SDE,max}}$
SEE	$\Delta U^{\text{SEE,nll}}$	[ ]	0.0425	Mörl et al. (2012)	relative stretch at non-linear linear transition
	$\Delta U^{\text{SEE,l}}$	[ ]	0.017	Mörl et al. (2012)	relative additional stretch in the linear part providing a force increase of $\Delta F^{\text{SEE},0}$
	$\Delta F^{\text{SEE},0}$	[N]	$0.4\,F^{\text{max}}$		both force at the transition and force increase in the linear part
Hatze	m	[1/s]	11.3	Kistemaker et al. (2006)	time constant for the activation dynamics
	c	[mol/l]	1.37e-4	Kistemaker et al. (2006)	constant for the activation dynamics
	η	[l/mol]	5.27e4	Kistemaker et al. (2006)	constant for the activation dynamics
	k	[ ]	2.9	Kistemaker et al. (2006)	constant for the activation dynamics
	q_0	[ ]	0.005	Günther (1997)	resting active state for all activated muscle fibers
	ν	[ ]	3	Kistemaker et al. (2006)	constant for the activation dynamics

Table 3: Muscle non-specific actuation parameters for the muscles and the activation dynamics.

Appendix

Mechanics parameters defining the geometry and the mechanical properties

```
% Gravity
PM.Gravity = [0 0 -9.80665];

%% %%%%%%%%%%%%%%%%%%%
% Segment parameters %
%%%%%%%%%%%%%%%%%%%%%%
PM.SegShoulder.p_Bone_CoM          = [0 0 0]; %[m] position of Bone CoM relative to proximal joint
PM.SegShoulder.p_joint_distal      = [0 -.1816 0]; %[m] position of distal joint relative to CoM

PM.SegShoulder.m_Bone              = 16.895560; %[kg] m
PM.SegShoulder.MomInert_Bone       = [0.096243 0.0811621 0.159251]; %[kg*m^2] Diagonal elements of the inertia tensor
PM.SegShoulder.ProdInert_Bone      = [0 0 0]; %[kg*m^2] Non-diagonal elements of the intertia tensor in the order [I_yz I_zx I_xy]
PM.SegUparm.p_Bone_CoM             = [0 0 0.146]*(-1); %[m] position of Bone CoM relative to proximal joint % *(-1) in comparison with demoa, source: Kistemaker2007
PM.SegUparm.p_joint_distal         = [0 0 -0.189]; %[m] position of distal joint relative to CoM

PM.SegUparm.m_Bone                 = 2.10; %[kg] mass, source: Kistemaker2007
PM.SegUparm.MomInert_Bone          = [0.0154388 0.024 0.00278951]; %[kg*m^2] Diagonal elements of the inertia tensor
PM.SegUparm.ProdInert_Bone         = [0 0 0]; %[kg*m^2] Non-diagonal elements of the intertia tensor in the order [I_yz I_zx I_xy]
PM.SegForearm.p_Bone_CoM           = [0 0 0.179]*(-1); %[m] position of Bone CoM relative to proximal joint, source: Kistemaker2007
PM.SegForearm.p_joint_distal       = [0 0 -0.084]; %[m] position of distal joint relative to CoM %Note: difference to demoa model, where: [0.015 0 -0.084]

PM.SegForearm.m_Bone               = 1.65; %[kg] mass, source: Kistemaker2007
PM.SegForearm.MomInert_Bone        = [0.009824518 0.025 0.001500813]; %[kg*m^2] Diagonal elements of the inertia tensor
PM.SegForearm.ProdInert_Bone       = [0 0 0]; %[kg*m^2] Non-diagonal elements of the intertia tensor in the order [I_yz I_zx I_xy]
PM.SegHand.p_Bone_CoM              = [0 0 0.07]*(-1); %[m] position of Bone CoM relative to proximal joint %Note: difference to demoa model, where: [0.015 0.0000 0.07]*(-1)
PM.SegHand.p_fingertip             = [0 0 -0.084]; %[m] position of distal joint relative to CoM

PM.SegHand.m_Bone                  = 0; %[kg] m
PM.SegHand.MomInert_Bone           = [0 0 0]; %[kg*m^2] Diagonal elements of the inertia tensor
PM.SegHand.ProdInert_Bone          = [0 0 0]; %[kg*m^2] Non-diagonal elements of the intertia tensor in the order [I_yz I_zx I_xy]

%%%%%%%%%%%%%%%%%%%%
% Deflection parameters %
%%%%%%%%%%%%%%%%%%%%

PM.Deflection.biart_flexor.rO                     = [0.03 -0.1816 0.02]; % Origin of the muscle relative to the center of mass of the parent body %Parent: Shoulder
PM.Deflection.biart_flexor.rI                     = [0.012, 0.0000, 0.12]; % Insertion of the muscle %Parent: Forearm
PM.Deflection.biart_flexor.Ellipse1.r             = [0.018, 0.0000, -0.1425]; % Coordinates of the reference point of deflection ellipse 1 relative to the parent body %Parent:Uparm
PM.Deflection.biart_flexor.Ellipse1.G             = [0 1 0]*0.0001; % Length of the half-axis of ellipse 1 in y direction
PM.Deflection.biart_flexor.Ellipse1.H             = [0 0 1]*0.0001; % % Length of the half-axis of ellipse 1 in z direction
PM.Deflection.biart_flexor.Ellipse1.angle         = [0,90,0]; % Angle [deg] of rotation of the ellipse triade around y-axis to orient the ellipse correctly
PM.Deflection.biart_flexor.Ellipse2.r             = [0.012, 0.0000, 0.125]; %Parent: Forearm
PM.Deflection.biart_flexor.Ellipse2.G             = [0 1 0]*0.0001;
PM.Deflection.biart_flexor.Ellipse2.H             = [0 0 1]*0.0001;
PM.Deflection.biart_flexor.Ellipse2.angle         = [0,0,0];

PM.Deflection.biart_extensor.rO                   = [-0.02 -0.1816 -0.03]; %Parent: Shoulder
PM.Deflection.biart_extensor.rI                   = [-0.0225, 0.0000, 0.1925]; %Parent: Forearm
PM.Deflection.biart_extensor.Ellipse1.r           = [-0.0225, 0.0000, -0.165]; %Parent:Uparm
PM.Deflection.biart_extensor.Ellipse1.G           = [0 1 0]*0.0001;
PM.Deflection.biart_extensor.Ellipse1.H           = [0 0 1]*0.00001;
PM.Deflection.biart_extensor.Ellipse1.angle       = [0,-90,0];
PM.Deflection.biart_extensor.Ellipse2.r           = [-0.0, 0.0000, 0.1975]; %Parent: Forearm
PM.Deflection.biart_extensor.Ellipse2.G           = [0 1 0]*0.0001;
PM.Deflection.biart_extensor.Ellipse2.H           = [0 0 1]*0.03;
PM.Deflection.biart_extensor.Ellipse2.angle       = [0,-60,0];

PM.Deflection.Shoulder_Anteversion.rO             = [0.00, -0.1816, 0.05]; %Parent: Shoulder
PM.Deflection.Shoulder_Anteversion.rI             = [0.01, 0.0000, 0.045];  %Parent:Uparm
PM.Deflection.Shoulder_Anteversion.Ellipse1.r     = [0.025, -0.1816, 0.04]; %Parent: Shoulder
PM.Deflection.Shoulder_Anteversion.Ellipse1.G     = [0 1 0]*0.0001;
PM.Deflection.Shoulder_Anteversion.Ellipse1.H     = [0 0 1]*0.0001;
PM.Deflection.Shoulder_Anteversion.Ellipse1.angle = [0,0,0];
PM.Deflection.Shoulder_Anteversion.Ellipse2.r     = [0.02, 0.0000, 0.1];  %Parent:Uparm
PM.Deflection.Shoulder_Anteversion.Ellipse2.G     = [0 1 0]*0.0001;
PM.Deflection.Shoulder_Anteversion.Ellipse2.H     = [0 0 1]*0.0001;
PM.Deflection.Shoulder_Anteversion.Ellipse2.angle = [0,90,0];

PM.Deflection.Shoulder_Retroversion.rO             = [-0.035, -0.1816, 0.045]; %Parent: Shoulder
PM.Deflection.Shoulder_Retroversion.rI             = [-0.01, 0.0000, 0.045];  %Parent:Uparm
PM.Deflection.Shoulder_Retroversion.Ellipse1.r     = [-0.04, -0.1816, -0.01]; %Parent: Shoulder
PM.Deflection.Shoulder_Retroversion.Ellipse1.G     = [0 1 0]*0.0001;
PM.Deflection.Shoulder_Retroversion.Ellipse1.H     = [0 0 1]*0.0001;
PM.Deflection.Shoulder_Retroversion.Ellipse1.angle = [0,0,0];
PM.Deflection.Shoulder_Retroversion.Ellipse2.r     = [-0.02, 0.0000, 0.1];  %Parent:Uparm
PM.Deflection.Shoulder_Retroversion.Ellipse2.G     = [0 1 0]*0.0001;
PM.Deflection.Shoulder_Retroversion.Ellipse2.H     = [0 0 1]*0.0001;
PM.Deflection.Shoulder_Retroversion.Ellipse2.angle = [0,90,0];

PM.Deflection.Elbow_flexor.rO                     = [0.01, 0.0000, 0.038]; %Parent:Uparm
PM.Deflection.Elbow_flexor.rI                     = [0.01, 0.0000, 0.12]; %Parent: Forearm
PM.Deflection.Elbow_flexor.Ellipse1.r             = [0.0, 0.0000, -0.132]; %Parent:Uparm
PM.Deflection.Elbow_flexor.Ellipse1.G             = [0 1 0]*0.0001;
PM.Deflection.Elbow_flexor.Ellipse1.H             = [0 0 1]*0.005;
```

```
PM.Deflection.Elbow_flexor.Ellipse1.angle          = [0,90,0];
PM.Deflection.Elbow_flexor.Ellipse2.r              = [0.01, 0.0000, 0.135]; %Parent: Forearm
PM.Deflection.Elbow_flexor.Ellipse2.G              = [0 1 0]*0.0001;
PM.Deflection.Elbow_flexor.Ellipse2.H              = [0 0 1]*0.003;
PM.Deflection.Elbow_flexor.Ellipse2.angle          = [0,90,0];

PM.Deflection.Elbow_extensor.rO                    = [-0.022, 0.0000, 0.0605]; %Parent:Uparm
PM.Deflection.Elbow_extensor.rI                    = [-0.0225, 0.0000, 0.1925]; %Parent: Forearm
PM.Deflection.Elbow_extensor.Ellipse1.r            = [-0.0225, 0.0000, -0.165]; %Parent:Uparm
PM.Deflection.Elbow_extensor.Ellipse1.G            = [0 1 0]*0.0001;
PM.Deflection.Elbow_extensor.Ellipse1.H            = [0 0 1]*0.00005;
PM.Deflection.Elbow_extensor.Ellipse1.angle        = [0,-90,0];
PM.Deflection.Elbow_extensor.Ellipse2.r            = [-0.0, 0.0000, 0.1975]; %Parent: Forearm
PM.Deflection.Elbow_extensor.Ellipse2.G            = [0 1 0]*0.0001;
PM.Deflection.Elbow_extensor.Ellipse2.H            = [0 0 1]*0.03;
PM.Deflection.Elbow_extensor.Ellipse2.angle        = [0,-60,0];
```

Listing 1: Mechanics parameters defining the geometry and the mechanical properties.

List of Abbreviations

MTU muscle-tendon unit

CE contractile element

PEE parallel elastic element

SEE serial elastic element

SDE serial damping element

EF Elbow Flexor

MEF Monarticular Elbow Flexor

EE Elbow Extensor

MEE Monarticular Elbow Extensor

BF Biarticular Flexor

BEFSA Biarticular Elbow Flexor Shoulder Anteversion

BE Biarticular Extensor

BEESR Biarticular Elbow Extensor Shoulder Retroversion

SF Shoulder Flexor

MSA Monoarticular Shoulder Anteversion

SE Shoulder Extensor

MSR Monoarticular Shoulder Retroversion

References

Aumüller, G., Aust, G., Engele, J., Kirsch, J., Maio, G., and Mayerhofer, A. *Duale Reihe Anatomie*. 2017. ISBN 9783132417526.

Bayer, A., Schmitt, S., Günther, M., and Haeufle, D. F. The influence of biophysical muscle properties on simulating fast human arm movements. *Computer Methods in Biomechanics and Biomedical Engineering*, 20(8):803–821, 2017. doi: 10.1080/10255842.2017.1293663.

Bhanpuri, N. H., Okamura, A. M., and Bastian, A. J. Predicting and correcting ataxia using a model of cerebellar function. *Brain*, 137(7):1931–1944, 2014. doi: 10.1093/brain/awu115.

Driess, D., Zimmermann, H., Wolfen, S., Suissa, D., Haeufle, D., Hennes, D., Toussaint, M., and Schmitt, S. Learning to Control Redundant Musculoskeletal Systems with Neural Networks and SQP: Exploiting Muscle Properties. *ICRA 2018 (accepted)*, 2018.

Günther, M. *Computersimulationen zur Synthetisierung des muskulär erzeugten menschlichen Gehens unter Verwendung eines biomechanischen Mehrkörpermodells*. PhD thesis, Eberhard-Karls-Universität zu Tübingen, 1997.

Haeufle, D. F. B., Günther, M., Bayer, A., and Schmitt, S. Hill-type muscle model with serial damping and eccentric force-velocity relation. *Journal of Biomechanics*, 47(6):1531–1536, 2014. doi: 10.1016/j.jbiomech.2014.02.009.

Hammer, M., Günther, M., Haeufle, D., and Schmitt, S. Tailoring anatomical muscle paths: a sheath-like solution for muscle routing in musculoskeletal computer models. *Mathematical Biosciences*, accepted (February):68–81, 2019. doi: 10.1016/j.mbs.2019.02.004.

Hatze, H. A Myocybernetic Control Model of Skeletal Muscle. *Biol. Cybernetics*, 25:103–119, 1977.

Kistemaker, D. a., Van Soest, A. J., and Bobbert, M. F. Is equilibrium point control feasible for fast goal-directed single-joint movements? *Journal of Neurophysiology*, 95(5):2898–912, 2006. doi: 10.1152/jn.00983.2005.

Kistemaker, D. A., Van Soest, A. J. K., Wong, J. D., Kurtzer, I., and Gribble, P. L. Control of position and movement is simplified by combined muscle spindle and Golgi tendon organ feedback. *Journal of Neurophysiology*, 109(4):1126–1139, 2013. doi: 10.1152/jn.00751.2012.

Mörl, F., Siebert, T., Schmitt, S., Blickhan, R., and Günther, M. Electro-Mechanical Delay in Hill-Type Muscle Models. *Journal of Mechanics in Medicine and Biology*, 12(05):1250085, 2012. doi: 10.1142/s0219519412500856.

Pigeon, P., Yahia, L., and Feldman, A. G. Moment arms and lengths of human upper limb muscles as functions of joint angles. *Journal of Biomechanics*, 29(10):1365–1370, 1996. doi: 10.1016/0021-9290(96)00031-0.

Sobotta, J. *Atlas der Anatomie des Menschen*, volume 3. Elsevier Health Sciences, 2010.

Suissa, D. R. *Modeling, Control and Optimization in Human Motor Control: A Simulation Study of a Physiological Human Arm*. Master thesis, University of Stuttgart, 2017.

van Soest, A., Bobbert, M., Iijima, T., Shimizu, K., and Asanuma, N. The Contribution of Muscle Properise in the Control of Explosive Movments. *Biological Cybernetics*, 69(3):195–204, 1993.
