# Supplementary material for: Predicting Perturbed Human Arm Movements in a Neuro-Musculoskeletal Model to Investigate the Muscular Force Response
Source: Front Bioeng Biotechnol. 2020 Apr 21;8:308. doi: 10.3389/fbioe.2020.00308 (PMC7186382; doi:10.3389/fbioe.2020.00308)
Supplement: Supplementary file 3 [file Data_Sheet_3.pdf]

# *Electronic supplementary material to "Predicting perturbed human arm movements in a neuro-musculoskeletal model to investigate the muscular force response": Additional results using time delay $\delta = 25\,\mathrm{ms}$*

Katrin Stollenmaier, Winfried Ilg, Daniel F.B. Haeufle
Department of Cognitive Neurology, Hertie Institute for Clinical Brain Research and
Werner Reichardt Centre for Integrative Neuroscience, University of Tübingen, Tübingen, Germany
Correspondence: Katrin Stollenmaier: katrin.stollenmaier@uni-tuebingen.de

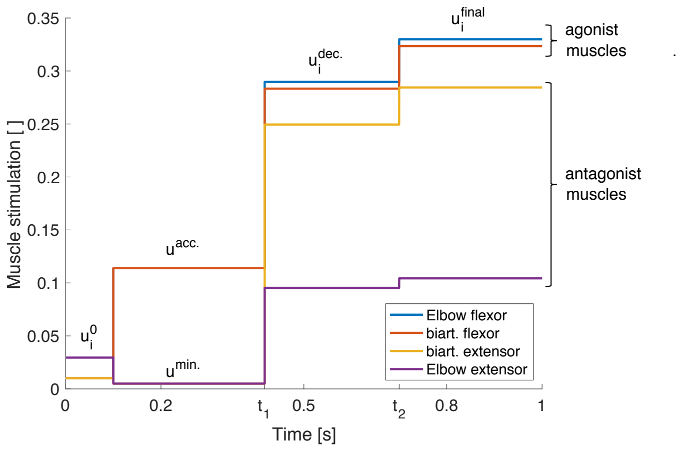


**Figure 1**: **Triphasic stimulation pattern for a flexion movement.** Starting from the initial position at $t = 0.1\,\mathrm{s}$, during the acceleration phase, mainly the agonist muscles are active. In the second phase between $t = t_1$ and $t = t_2$, both muscle groups are active, braking the movement. In the last phase for $t > t_2$, again both muscle groups are active in order to reach the final position and hold it with a desired level of co-contraction.

# 1 RESULTS

## 1.1 Static perturbation of inertia and viscosity

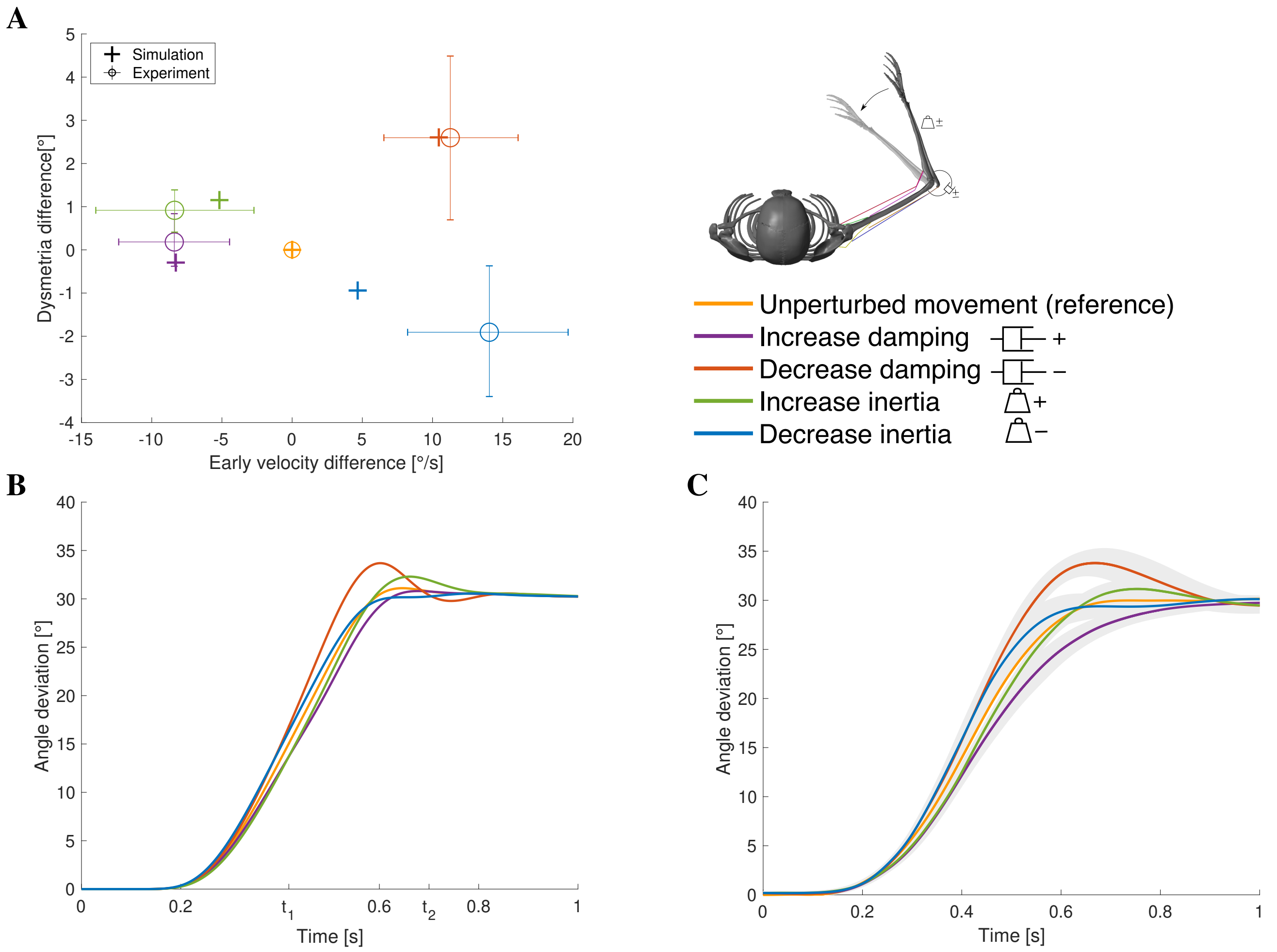


**Figure 2**: **Results for case ①**. **A** Evaluation criterion for the static perturbations: Early velocity difference in relation to the dysmetria difference (both calculated as the early velocity/dysmetria of the perturbed movement minus the early velocity/dysmetria of the reference movement) shown for both, simulation and experiment. The experimental results are digitized from Bhanpuri et al. (2014), the control group averages (n=11) are shown and the error bars indicate standard deviation. **B** our simulation results and **C** experimental results digitized from Bhanpuri et al. (2014) for one typical control subject in null condition (reference) and with perturbations (shaded areas indicate standard deviation).

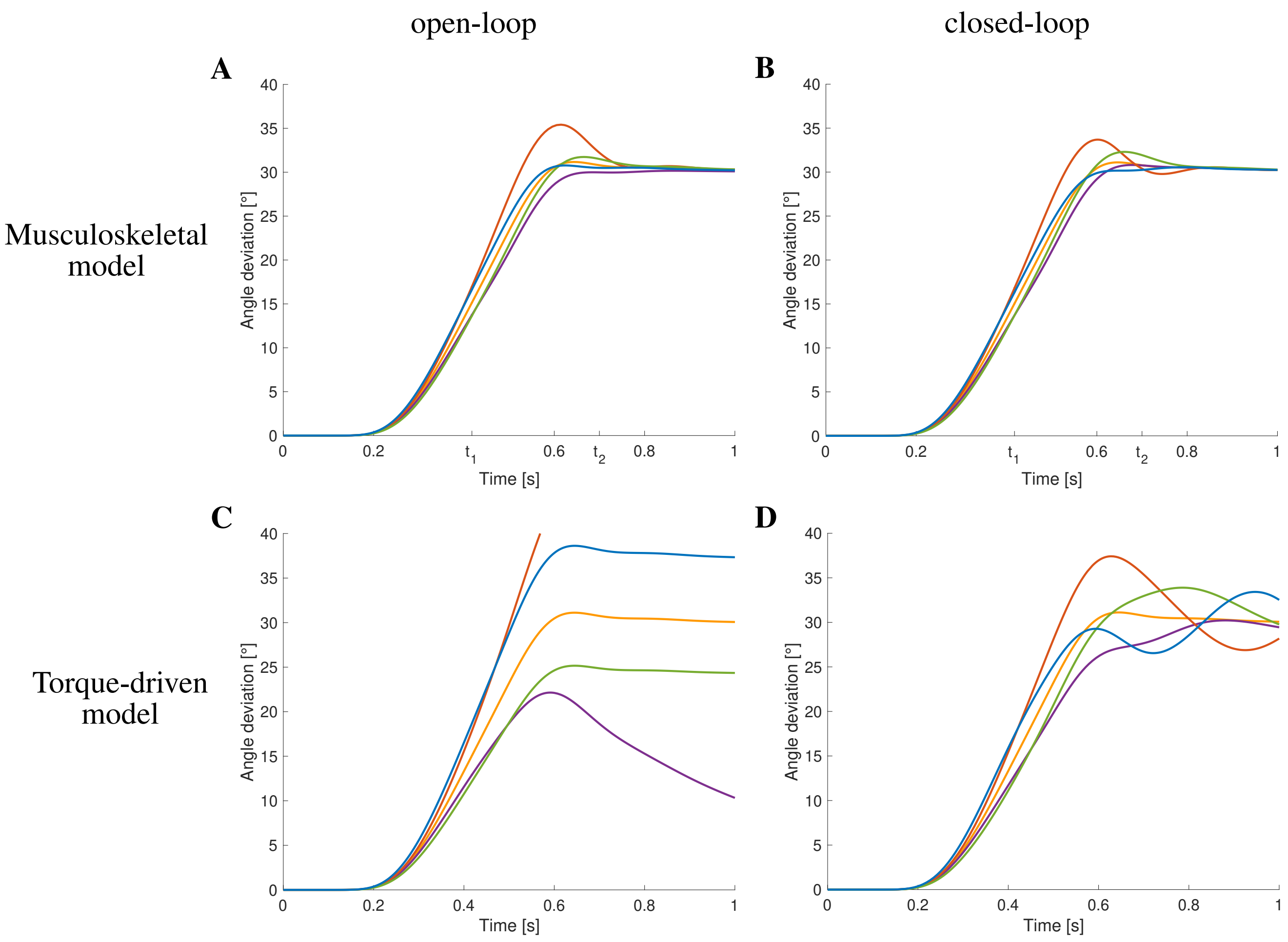


**Figure 3**: **Comparison to open-loop and torque-driven model for case ①.** **A** Resulting trajectories when controlling the musculoskeletal model open-loop, **B** trajectories when controlling the musculoskeletal model closed-loop, **C** trajectories when controlling a purely torque-driven model open-loop and **D** trajectories when controlling a purely torque-driven model closed-loop.

| | Closed-loop | Open-loop |
|---|---|---|
| Increased damping | 0.53 | 3.47 |
| Decreased damping | 0.03 | 0.70 |
| Increased inertia | 0.54 | 0.84 |
| Decreased inertia | 2.99 | 2.94 |
| Sum of all cases | 4.09 | 7.94 |

**Table 1.** Quantification of the difference between simulation and experiment for case ① by evaluating the cost function that was used in the optimization of the closed-loop control parameters and splitting it into the contributions of the different perturbations. Hence, for the single cases, a value smaller than one means that the result lies within the experimental standard deviation area (taking the maximum standard deviation in each direction).

## 1.2 Dynamic torque perturbation

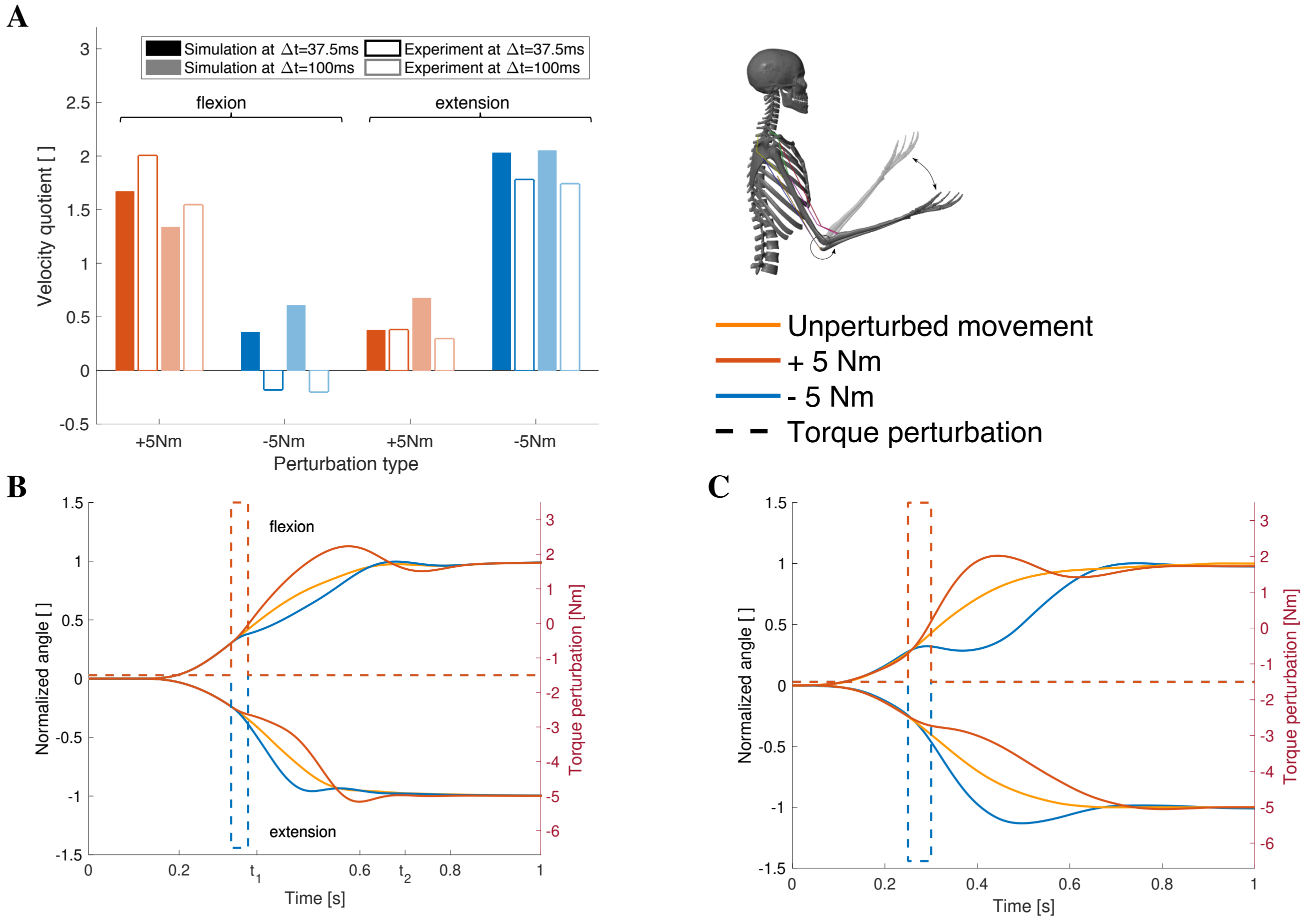


**Figure 4**: **Results for case ②**. **A** Evaluation criterion for the dynamic perturbations: The quotient of the angular velocity at the beginning the perturbation and after $\Delta t$ ($37.5\,\mathrm{ms}$ and $100\,\mathrm{ms}$) shown for both, the simulation results (filled bars) and the experimental results (empty bars) for all four perturbation types (experimental results are digitized from Kalveram and Seyfarth (2009)). **B** Joint angle trajectories for the four different perturbation types in our simulation and **C** in the experiment (digitized from Kalveram and Seyfarth (2009)). Note that the experimental results show the trajectory for one typical control subject. The upper curves show flexion movements, the lower curves show extension movements. The dashed lines visualize the applied torque perturbations.

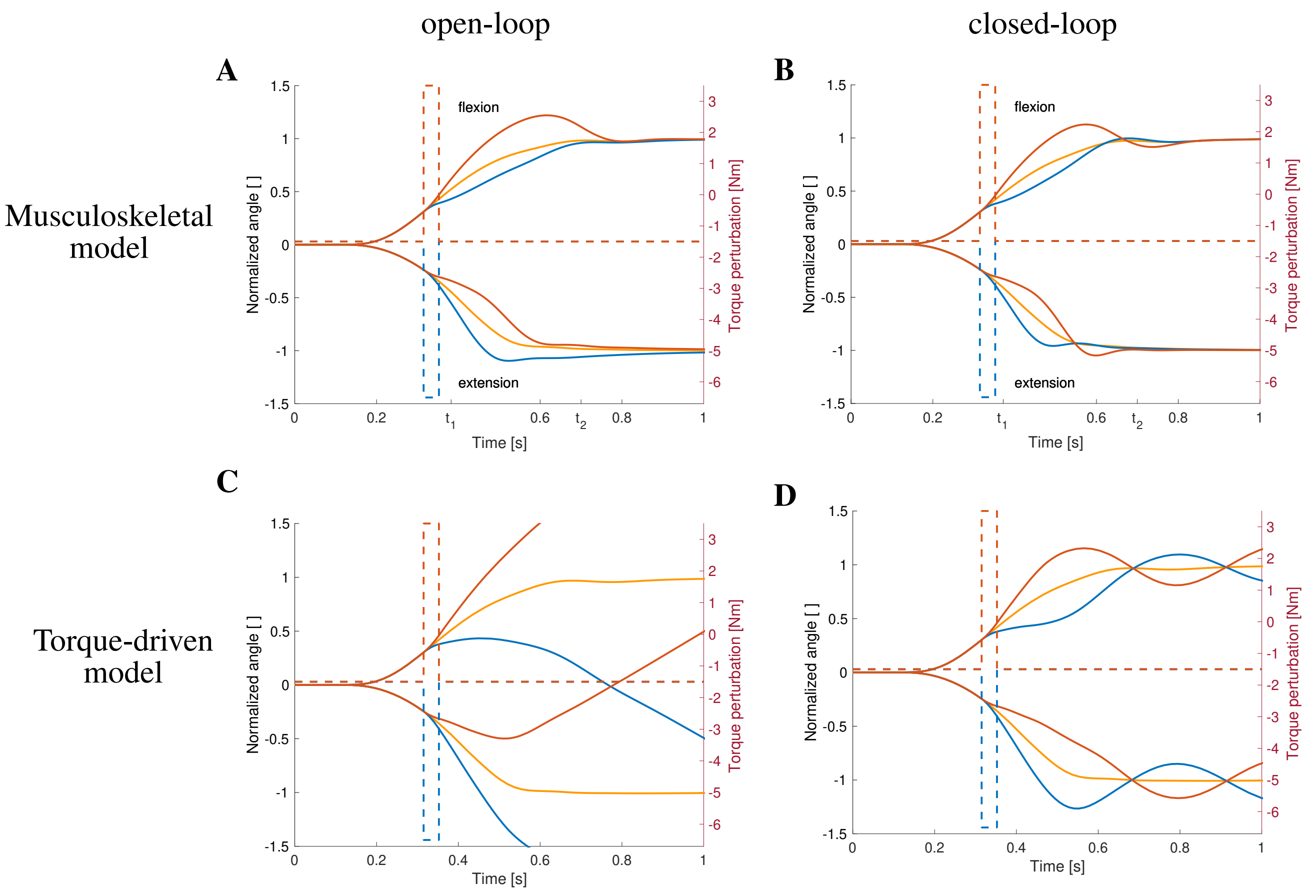


**Figure 5**: **Comparison to open-loop and torque-driven model for case ②. A** Resulting trajectories when controlling the musculoskeletal model open-loop, **B** trajectories when controlling the musculoskeletal model closed-loop, **C** trajectories when controlling a purely torque-driven model open-loop and **D** trajectories when controlling a purely torque-driven model closed-loop with the same controller as described above.

## 1.3 Internal force responses

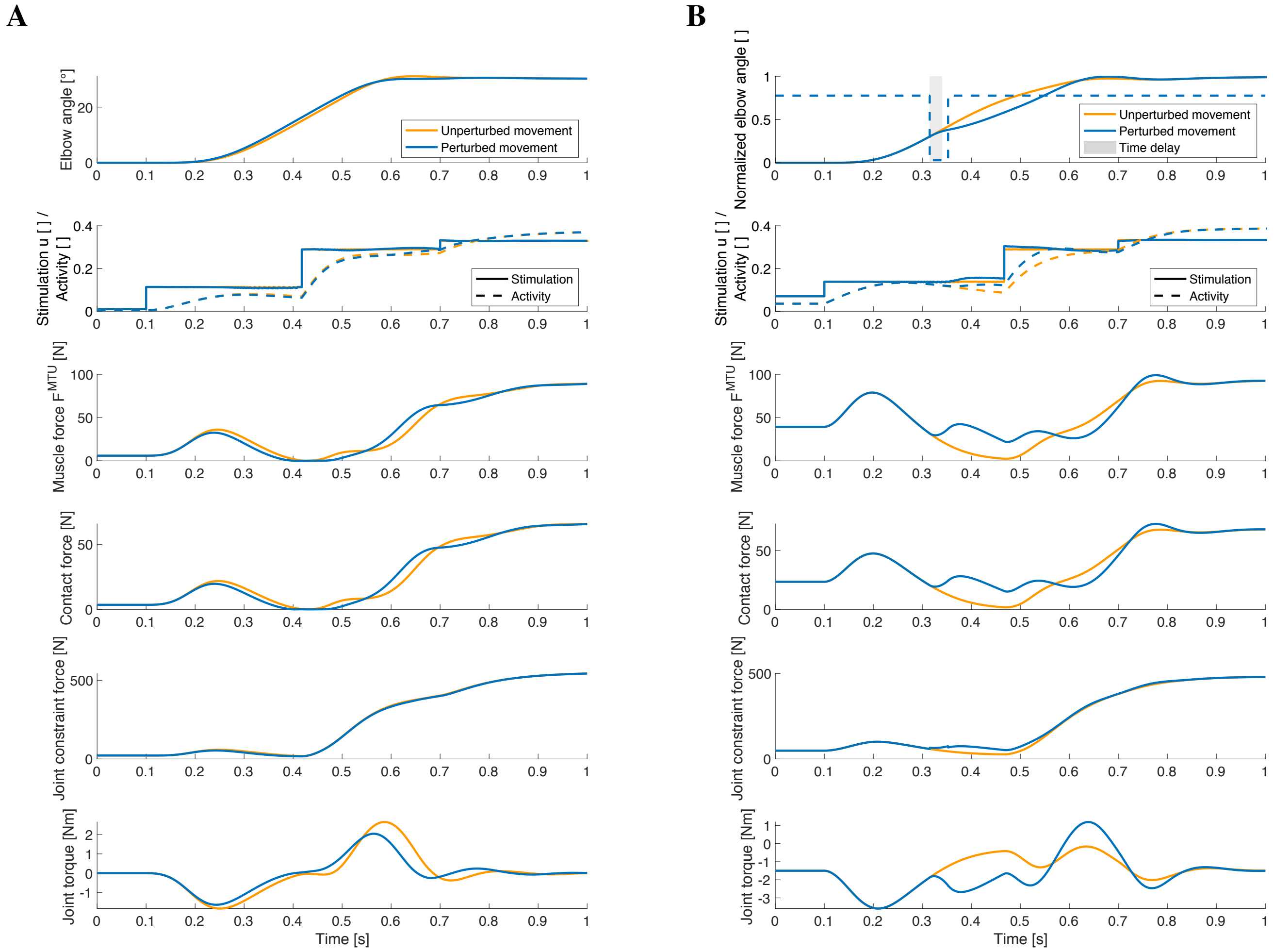


**Figure 6**: **Selection of quantities that can be investigated using our model.** Elbow joint angle, muscle stimulation and activity, muscle force, muscle-bone contact force, joint constraint force and active joint torque for the unperturbed trajectory (orange) and for a perturbed movement (blue). These results are exemplary shown for the elbow flexor muscle and **A** for an increase in inertia and **B** for a flexion movement with a negative torque impulse perturbation. Here, the gray area visualizes the length of the time delay in the controller ($25\,\mathrm{ms}$), i.e. the time after the perturbation before the feedback mechanism is activated. Note that the total muscle stimulation in the unperturbed case is equal to the open-loop contribution in the perturbed case. For all forces, the resultant force is shown. The contact force is the force at the first deflection ellipse (positions of the ellipses see Supplementary Material). The active joint torque represents the torque acting on the joint that is a consequence of the muscle forces.

# APPENDIX

| | | | ① without external torque | ② with external torque, flexion | ② with external torque, extension |
|---|---|---|---|---|---|
| initial position | $u^{\text{des.},0}$ | | $0^*$ | $0^*$ | $0^*$ |
| | $u_i^0$ | Elbow flexor | $0.0100^{\#}$ | $0.0704^{\#}$ | $0.198^{\#}$ |
| | | Elbow extensor | $0.0295^{\#}$ | $0.0101^{\#}$ | $0.0165^{\#}$ |
| | | biart. flexor | $0.0101^{\#}$ | $0.0101^{\#}$ | $0.0539^{\#}$ |
| | | biart. extensor | $0.0101^{\#}$ | $0.0101^{\#}$ | $0.0286^{\#}$ |
| accele-ration | $u^{\text{min.}}$ | | $0.005^*$ | $0.005^*$ | $0.005^*$ |
| | $u^{\text{acc.}}$ | | $0.114^{\triangle}$ | $0.138^{\triangle}$ | $2.51 \times 10^{-6\triangle}$ |
| | $t_1$ | | $0.418\,\text{s}^{\triangle}$ | $0.467\,\text{s}^{\triangle}$ | $0.372\,\text{s}^{\triangle}$ |
| decele-ration | $u^{\text{des.,dec.}}$ | | $0.262^{\diamondsuit}$ | $0.262^{\diamondsuit}$ | $0.262^{\diamondsuit}$ |
| | $u_i^{\text{dec.}}$ | Elbow flexor | $0.290^{\#}$ | $0.290^{\#}$ | $0.212^{\#}$ |
| | | Elbow extensor | $0.0954^{\#}$ | $0.0954^{\#}$ | $0.283^{\#}$ |
| | | biart. flexor | $0.283^{\#}$ | $0.283^{\#}$ | $0.237^{\#}$ |
| | | biart. extensor | $0.249^{\#}$ | $0.249^{\#}$ | $0.266^{\#}$ |
| | $t_2$ | | $0.7\,\text{s}^*$ | $0.7\,\text{s}^*$ | $0.7\,\text{s}^*$ |
| final position | $u^{\text{des.,final}}$ | | $0.3^*$ | $0.3^*$ | $0.3^*$ |
| | $u_i^{\text{final}}$ | Elbow flexor | $0.330^{\#}$ | $0.334^{\#}$ | $0.221^{\#}$ |
| | | Elbow extensor | $0.104^{\#}$ | $0.0928^{\#}$ | $0.325^{\#}$ |
| | | biart. flexor | $0.324^{\#}$ | $0.326^{\#}$ | $0.266^{\#}$ |
| | | biart. extensor | $0.284^{\#}$ | $0.283^{\#}$ | $0.307^{\#}$ |
| | $k_p$ | | $0.503^{\diamondsuit}$ | $0.503^{\diamondsuit}$ | $0.503^{\diamondsuit}$ |
| | $k_d$ | | $0.0484^{\diamondsuit}$ | $0.0484^{\diamondsuit}$ | $0.0484^{\diamondsuit}$ |
| | $\delta$ | | $0.025^*$ | $0.025^*$ | $0.025^*$ |

**Table 2.** Control parameters used in the computer simulation of external perturbations during point-to-point movements in a horizontal plane. For a better understanding of the abbreviations see Figure 1. Gray values indicate that the same values as for ① have been used. Meaning of the symbols: $*$: quantities that we set to a fixed value, $\#$: optimized such that there is an equilibrium point at this position given the desired level of co-contraction, $\triangle$: optimized to match the unperturbed trajectory for case ①, $\diamondsuit$: optimized to match the perturbed trajectories for case ①.

## REFERENCES

Bhanpuri, N. H., Okamura, A. M., and Bastian, A. J. (2014). Predicting and correcting ataxia using a model of cerebellar function. *Brain* 137, 1931–1944

Kalveram, K. T. and Seyfarth, A. (2009). Inverse biomimetics: How robots can help to verify concepts concerning sensorimotor control of human arm and leg movements. *Journal of Physiology Paris* 103, 232–243
